# Supplementary figures and images for: In-vitro evaluation of probiotic potential of gut microbes isolated from retail chicken
Source: PLoS One. 2026 Jan 28;21(1):e0340981. doi: 10.1371/journal.pone.0340981 (PMC12851499; doi:10.1371/journal.pone.0340981)

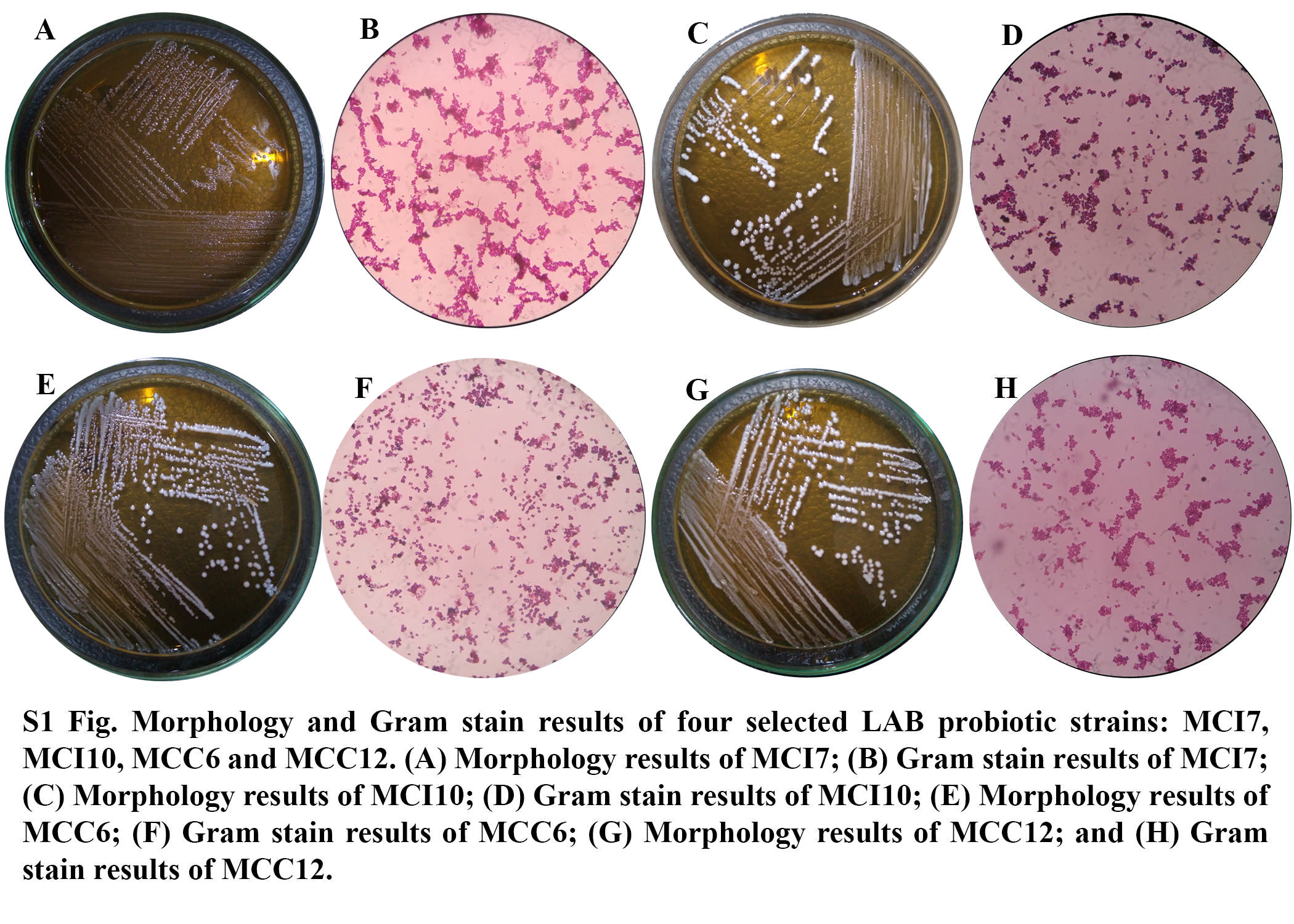

Supplement: S1 Fig — (A) Morphology results of MCI7; (B) Gram stain results of MCI7; (C) Morphology results of MCI10; (D) Gram stain results of MCI10; (E) Morphology results of MCC6; (F) Gram stain results of MCC6; (G) Morphology results of MCC12; and (H) Gram stain results of MCC12. (TIFF) [file pone.0340981.s001.tiff]

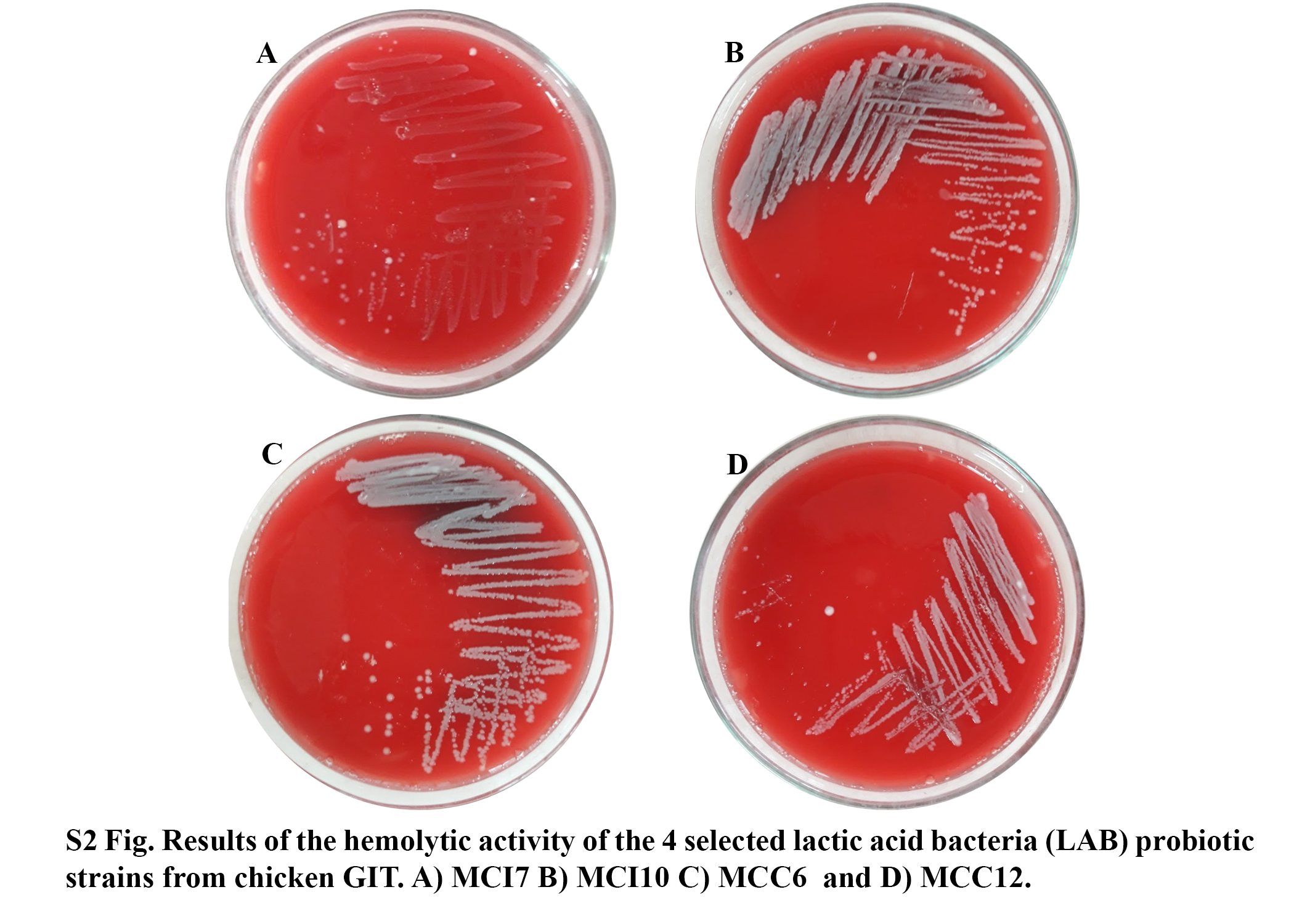

Supplement: S2 Fig — A) MCI7 B) MCI10 C) MCC6 and D) MCC12. (TIF) [file pone.0340981.s002.tif]

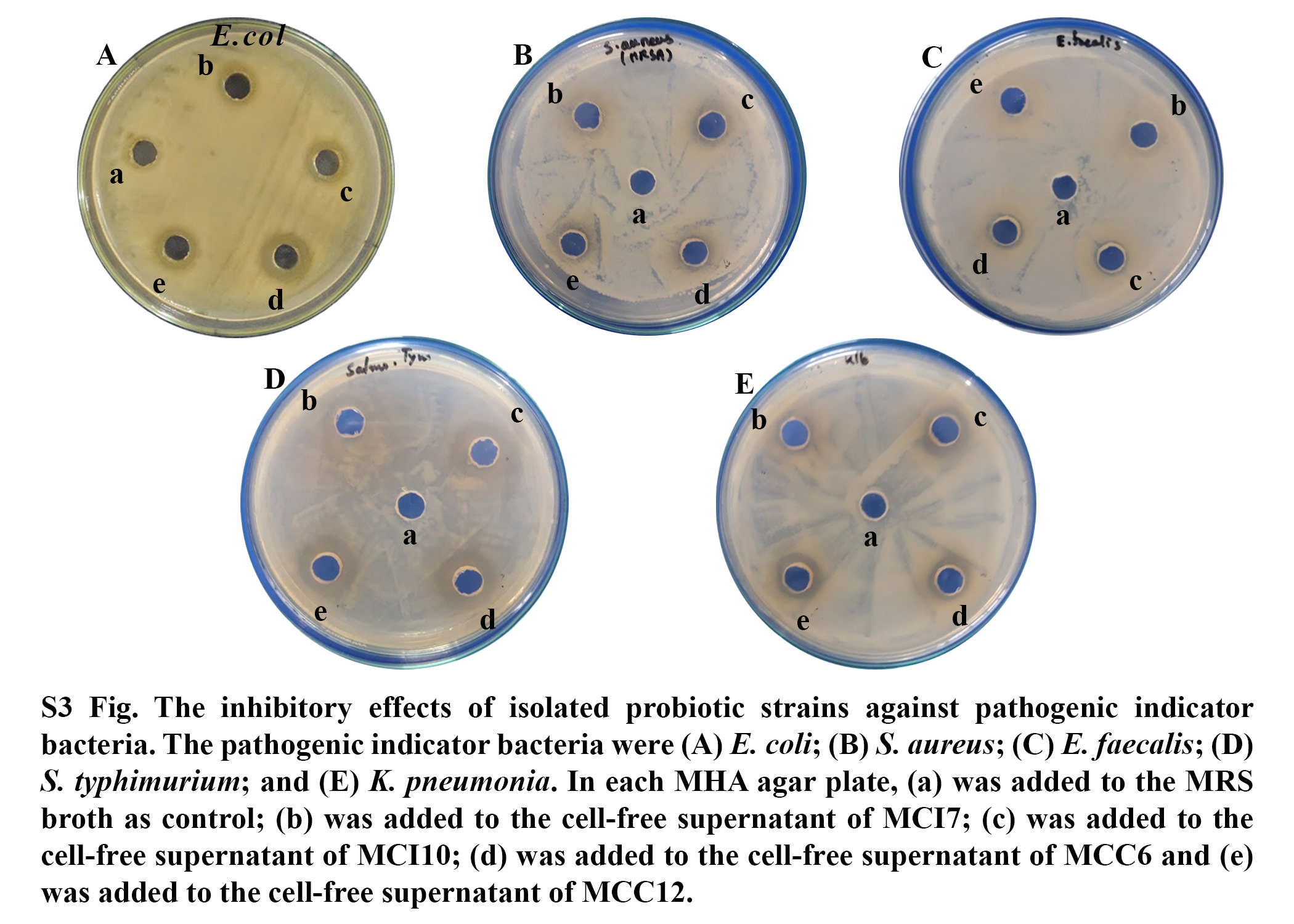

Supplement: S3 Fig — The pathogenic indicator bacteria were (A) E. coli; (B) S. aureus; (C) E. faecalis; (D) S. typhimurium; and (E) K. pneumonia. In each MHA agar plate, (a) was added to the MRS broth as control; (b) was added to the cell-free supernatant of MCI7; (c) was added to the cell-free supernatant of MCI10; (d) was added to the cell-free supernatant of MCC6 and (e) was added to the cell-free supernatant of MCC12. (TIFF) [file pone.0340981.s003.tiff]

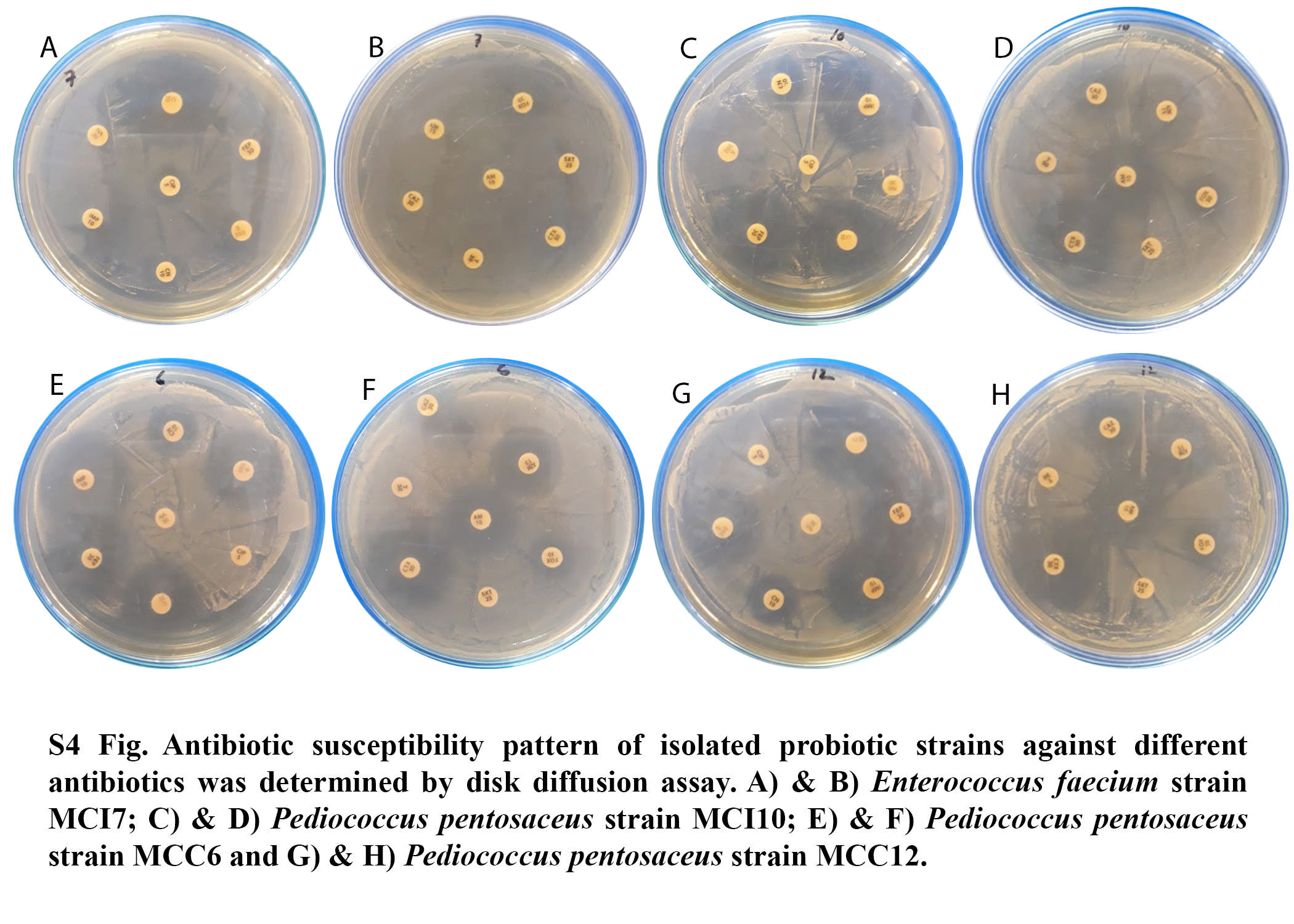

Supplement: S4 Fig — A) & B) Enterococcus faecium strain MCI7; C) & D) Pediococcus pentosaceus strain MCI10; E) & F) Pediococcus pentosaceus strain MCC6 and G) & H) Pediococcus pentosaceus strain MCC12. (TIFF) [file pone.0340981.s004.tiff]

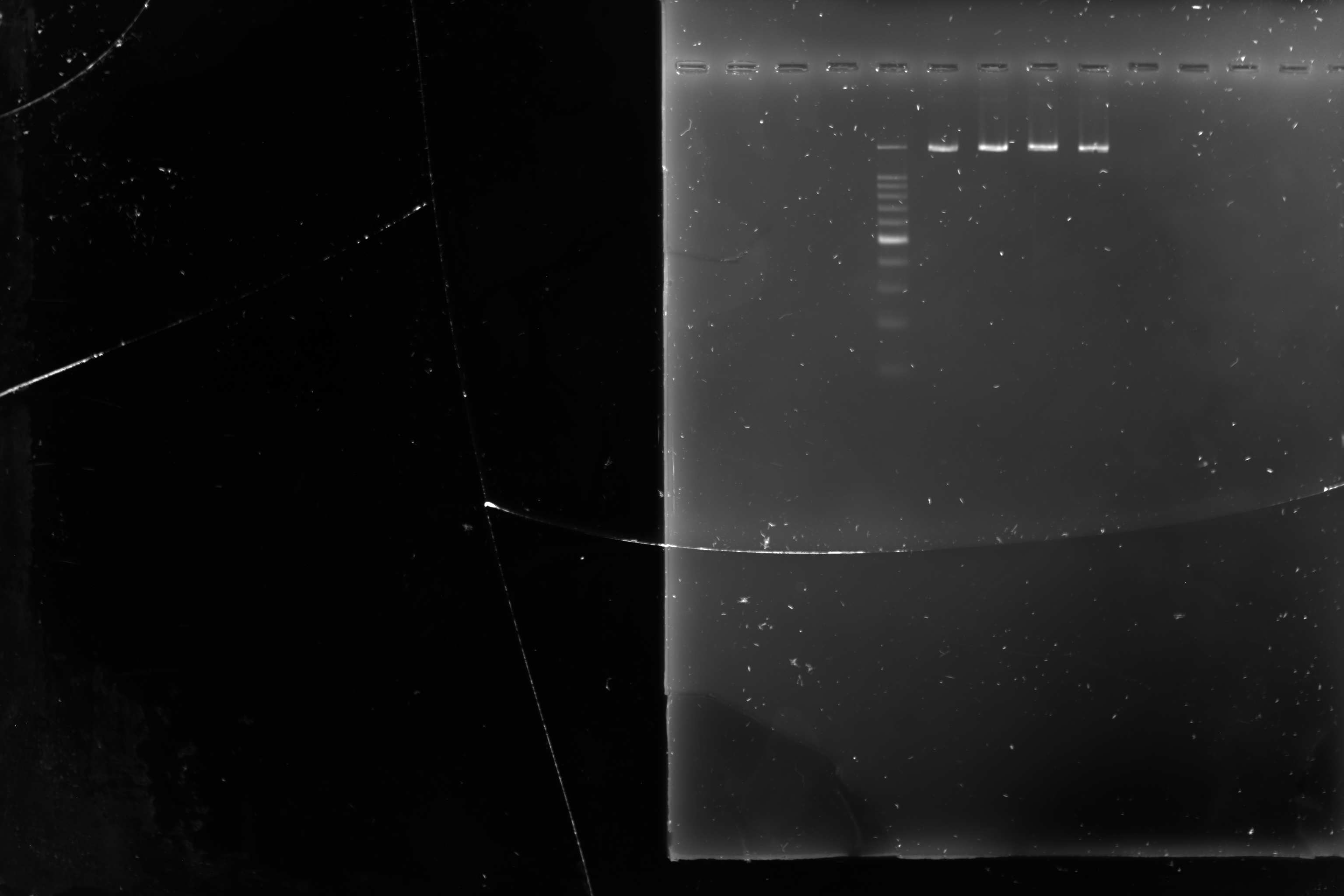

Supplement: S1 File — (TIF) [file pone.0340981.s008.tif]
