# Supplementary material for: In-vitro evaluation of probiotic potential of gut microbes isolated from retail chicken
Source: PLoS One. 2026 Jan 28;21(1):e0340981. doi: 10.1371/journal.pone.0340981 (PMC12851499; doi:10.1371/journal.pone.0340981)
Supplement: S1 Table — (DOCX) [file pone.0340981.s005.docx]

**S1 Table.** **Hemolytic activities of potential lactic acid bacteria (LAB) probiotic strains from chicken GIT.**

| **Isolate** | **Type of Hemolysis** |
| --- | --- |
| MCI2 | ϒ-hemolysis |
| MCI7 | ϒ-hemolysis |
| MCI10 | ϒ-hemolysis |
| MCI11 | ϒ-hemolysis |
| MCC6 | ϒ-hemolysis |
| MCC10 | ϒ-hemolysis |
| MCC12 | ϒ-hemolysis |
