# Supplementary material for: In-vitro evaluation of probiotic potential of gut microbes isolated from retail chicken
Source: PLoS One. 2026 Jan 28;21(1):e0340981. doi: 10.1371/journal.pone.0340981 (PMC12851499; doi:10.1371/journal.pone.0340981)
Supplement: S2 Table — (DOCX) [file pone.0340981.s006.docx]

**S2** Table. Survival of potential lactic acid bacteria (LAB) probiotic strains at 2, 4, and 6% NaCl concentration estimated through measuring OD^600^.

| **Isolates** | **OD (600 nm) at different concentration of NaCl after 24 h** | | | | | | | | | | | | | | | | | |
| --- | --- | --- | --- | --- | --- | --- | --- | --- | --- | --- | --- | --- | --- | --- | --- | --- | --- | --- |
|  | **2% NaCl** | | | | | | **4% NaCl** | | | | | | **6% NaCl** | | | | | |
|  | **1^st^** | **2^nd^** | **3^rd^** | **Mean** | **SD** | **p-value** | **1^st^** | **2^nd^** | **3^rd^** | **Mean** | **SD** | **p-value** | **1^st^** | **2^nd^** | **3^rd^** | **Mean** | **SD** | **p-value** |
| **MCI2** | 0.933 | 0.875 | 0.865 | 0.891 | ± 0.03 | **<0.001** | 0.56 | 0.515 | 0.53 | 0.535 | ± 0.02 | **<0.001** | 0.121 | 0.154 | 0.095 | 0.123 | ± 0.02 | **<0.001** |
| **MCI7** | 1.175 | 1.186 | 1.148 | 1.169 | ± 0.02 |  | 0.937 | 0.925 | 0.906 | 0.922 | ± 0.01 |  | 0.816 | 0.789 | 0.808 | 0.804 | ± 0.01 |  |
| **MCI10** | 1.287 | 1.257 | 1.266 | 1.27 | ± 0.01 |  | 1.18 | 1.151 | 1.167 | 1.166 | ± 0.01 |  | 1.115 | 1.084 | 1.053 | 1.084 | ± 0.03 |  |
| **MCI11** | 0.539 | 0.601 | 0.51 | 0.55 | ± 0.04 |  | 0.301 | 0.288 | 0.27 | 0.286 | ± 0.01 |  | 0.078 | 0.067 | 0.09 | 0.078 | ± 0.01 |  |
| **MCC6** | 1.733 | 1.654 | 1.677 | 1.688 | ± 0.04 |  | 1.57 | 1.533 | 1.516 | 1.539 | ± 0.03 |  | 1.457 | 1.435 | 1.373 | 1.421 | ± 0.04 |  |
| **MCC10** | 1.338 | 1.283 | 1.316 | 1.312 | ± 0.02 |  | 0.953 | 0.992 | 0.978 | 0.974 | ± 0.02 |  | 0.872 | 0.827 | 0.846 | 0.848 | ± 0.02 |  |
| **MCC12** | 1.613 | 1.576 | 1.63 | 1.606 | ± 0.02 |  | 1.367 | 1.298 | 1.331 | 1.332 | ± 0.03 |  | 0.985 | 1.015 | 1.067 | 1.022 | ± 0.04 |  |

Values are significantly different (P < 0.05) at 5% level of probability.
