# Supplementary material for: In-vitro evaluation of probiotic potential of gut microbes isolated from retail chicken
Source: PLoS One. 2026 Jan 28;21(1):e0340981. doi: 10.1371/journal.pone.0340981 (PMC12851499; doi:10.1371/journal.pone.0340981)
Supplement: S3 Table — (DOCX) [file pone.0340981.s007.docx]

**S3 Table. Bile salt resistance of potential lactic acid bacteria (LAB) probiotic strains, estimated by measuring OD^600^ in the presence and absence of bile salts.**

| **Isolates** | **OD of control after 4h incubation (600nm)** | | | | | | **OD with 0.3% bile salt after 4h incubation (600nm)** | | | | | |
| --- | --- | --- | --- | --- | --- | --- | --- | --- | --- | --- | --- | --- |
|  | **1^st^** | **2^nd^** | **3^rd^** | **Mean** | **SD** | **p-value** | **1^st^** | **2^nd^** | **3^rd^** | **Mean** | **SD** | **p-value** |
| **MCI7** | 0.408 | 0.351 | 0.379 | 0.379 | ± 0.02 | **<0.001** | 0.283 | 0.245 | 0.237 | 0.255 | ± 0.02 | **<0.001** |
| **MCI10** | 0.546 | 0.528 | 0.507 | 0.527 | ± 0.01 |  | 0.413 | 0.369 | 0.388 | 0.390 | ± 0.02 |  |
| **MCC6** | 0.582 | 0.547 | 0.555 | 0.561 | ± 0.01 |  | 0.452 | 0.436 | 0.427 | 0.438 | ± 0.01 |  |
| **MCC12** | 0.601 | 0.573 | 0.541 | 0.571 | 0.03 |  | 0.427 | 0.416 | 0.401 | 0.415 | ± 0.01 |  |

Values are significantly different (P < 0.05) at 5% level of probability.
