# Supplementary material for: In-vitro evaluation of probiotic potential of gut microbes isolated from retail chicken
Source: PLoS One. 2026 Jan 28;21(1):e0340981. doi: 10.1371/journal.pone.0340981 (PMC12851499; doi:10.1371/journal.pone.0340981)
Supplement: Attachment — Submitted filename: PONE-D-25-29338 revised.pdf [file pone.0340981.s009.pdf]

PLOS ONE

In-vitro evaluation of probiotic potential of gut microbes isolated from retail chicken.

--Manuscript Draft--

**Manuscript Number:**	PONE-D-25-29338
**Article Type:**	Research Article
**Full Title:**	In-vitro evaluation of probiotic potential of gut microbes isolated from retail chicken.
**Short Title:**	In-vitro evaluation of chicken gut microbes for probiotic potential.
**Corresponding Author:**	Sabina Yasmin, PhD<br>Bangladesh Agricultural University<br>Mymensingh, BANGLADESH
**Keywords:**	lactic acid bacteria; antagonistic activity; bile salts; GIT; hemolysis
**Abstract:**	Probiotics are live, non-pathogenic microorganisms that help to improve the host's gut health when administrated in sufficient proportions and are now serving as effective alternatives to antibiotics for managing animal infections and enhancing production. The objective of this study was to isolate, identify and characterize lactic acid bacteria (LAB) strains with excellent probiotic properties from the gastrointestinal tract (GIT) of retail broiler chickens. Samples were enriched in MRS broth at 37°C and plated on MRS agar to isolate distinct colonies of potential probiotic candidates. The isolates underwent a series of standard morphological and biochemical analysis to fulfill the criteria for probiotic characteristics. These analyses included Gram staining, catalase testing, hemolytic activity assays, tolerance assays to NaCl, low pH and bile salts, antagonistic activity assays, antibiotic susceptibility testing, and genotypic identification through 16S rRNA gene sequencing. A total of 40 microbial strains were isolated from the GIT of 20 retail broiler chickens. Of these, 4 LAB strains showed the best probiotic results and were genotypically identified as Enterococcus faecium MCI7, Pedicoccus pentosaceus MCI10, Pediococcus pentosaceus MCC6 and Pediococcus pentosaceus MCC12. The selected strains exhibited non-hemolytic activity and demonstrated excellent survival in an acidic environment with a pH level of 3. Furthermore, the strains displayed bile salt suppression in the presence of 0.3% bile salt for 4 hours, ranging from 21.91 to 32.77% and a wide range of antimicrobial activities against various pathogenic bacterial strains with inhibition zones ranging from 10 to 16.5 mm. Moreover, P. pentosaceus MCI10, P. pentosaceus MCC6 and P. pentosaceus MCC12 were sensitive to the majority of the tested antibiotics. Our study identified four LAB strains as promising probiotic candidates for poultry feed additives to effectively establish intestinal microflora, enhance meat quality and growth, and control pathogens.
**Order of Authors:**	Sangram Biswas, MS
	Lutfor Rahman, MS
	Susmita Chowdhury
	Fahmida Khatun, PhD
	Azimun Nahar, PhD
	Sabina Yasmin, PhD
**Additional Information:**	
**Question**	**Response**
**Financial Disclosure**<br><br>Enter a financial disclosure statement that describes the sources of funding for the work included in this submission. Review the submission guidelines for detailed requirements. View published research	The author(s) received no specific funding for this work.

Powered by Editorial Manager® and ProduXion Manager® from Aries Systems Corporation

articles from *PLOS ONE* for specific examples.<br><br>This statement is required for submission and **will appear in the published article** if the submission is accepted. Please make sure it is accurate.<br><br>**Funded studies**<br>Enter a statement with the following details:<br>• Initials of the authors who received each award<br>• Grant numbers awarded to each author<br>• The full name of each funder<br>• URL of each funder website<br>• Did the sponsors or funders play any role in the study design, data collection and analysis, decision to publish, or preparation of the manuscript?<br><br>Did you receive funding for this work?	
**Competing Interests**<br><br>Use the instructions below to enter a competing interest statement for this submission. On behalf of all authors, disclose any competing interests that could be perceived to bias this work—acknowledging all financial support and any other relevant financial or non-financial competing interests.<br><br>This statement is required for submission and **will appear in the published article** if the submission is accepted. Please make sure it is accurate and that any funding sources listed in your Funding Information later in the submission form are also declared in your Financial Disclosure statement.<br><br>View published research articles from *PLOS ONE* for specific examples.	The authors have declared that no competing interests exist.

**NO authors have competing interests**<br><br>Enter: *The authors have declared that no competing interests exist.*<br><br>**Authors with competing interests**<br><br>Enter competing interest details beginning with this statement:<br><br>*I have read the journal's policy and the authors of this manuscript have the following competing interests: [insert competing interests here]*<br><br>* typeset	
**Ethics Statement**<br><br>Enter an ethics statement for this submission. This statement is required if the study involved:<br><br>• Human participants<br>• Human specimens or tissue<br>• Vertebrate animals or cephalopods<br>• Vertebrate embryos or tissues<br>• Field research<br><br>Write "N/A" if the submission does not require an ethics statement.<br><br>General guidance is provided below. Consult the submission guidelines for detailed instructions. **Make sure that all information entered here is included in the Methods section of the manuscript.**	: The animal study protocol was approved by the Animal Welfare and Experimentation Ethics Committee, Bangladesh Agricultural University, Mymensingh-2202, Bangladesh (AWEEC/BAU/2025(2)/32(a), Date: 25-05-2025).

Powered by Editorial Manager® and ProduXion Manager® from Aries Systems Corporation

**Format for specific study types**<br><br>**Human Subject Research (involving human participants and/or tissue)**<br>• Give the name of the institutional review board or ethics committee that approved the study<br>• Include the approval number and/or a statement indicating approval of this research<br>• Indicate the form of consent obtained (written/oral) or the reason that consent was not obtained (e.g. the data were analyzed anonymously)<br><br>**Animal Research (involving vertebrate animals, embryos or tissues)**<br>• Provide the name of the Institutional Animal Care and Use Committee (IACUC) or other relevant ethics board that reviewed the study protocol, and indicate whether they approved this research or granted a formal waiver of ethical approval<br>• Include an approval number if one was obtained<br>• If the study involved *non-human primates*, add *additional details* about animal welfare and steps taken to ameliorate suffering<br>• If anesthesia, euthanasia, or any kind of animal sacrifice is part of the study, include briefly which substances and/or methods were applied<br><br>**Field Research**<br><br>Include the following details if this study involves the collection of plant, animal, or other materials from a natural setting:<br>• Field permit number<br>• Name of the institution or relevant body that granted permission	
**Data Availability**<br><br>Authors are required to make all data underlying the findings described fully available, without restriction, and from the time of publication. PLOS allows rare exceptions to address legal and ethical concerns. See the PLOS Data Policy and FAQ for detailed information.	Yes - all data are fully available without restriction

Powered by Editorial Manager® and ProduXion Manager® from Aries Systems Corporation

A Data Availability Statement describing where the data can be found is required at submission. Your answers to this question constitute the Data Availability Statement and **will be published in the article**, if accepted.<br><br>**Important:** Stating 'data available on request from the author' is not sufficient. If your data are only available upon request, select 'No' for the first question and explain your exceptional situation in the text box.<br><br>Do the authors confirm that all data underlying the findings described in their manuscript are fully available without restriction?	
**Describe where the data may be found in full sentences. If you are copying our sample text, replace any instances of XXX with the appropriate details.**<br><br>• If the data are **held or will be held in a public repository**, include URLs, accession numbers or DOIs. If this information will only be available after acceptance, indicate this by ticking the box below. For example: *All XXX files are available from the XXX database (accession number(s) XXX, XXX.*).<br>• If the data are all contained **within the manuscript and/or Supporting Information files**, enter the following: *All relevant data are within the manuscript and its Supporting Information files.*<br>• If neither of these applies but you are able to provide **details of access elsewhere**, with or without limitations, please do so. For example:<br><br>*Data cannot be shared publicly because of [XXX]. Data are available from the XXX Institutional Data Access / Ethics Committee (contact via XXX) for researchers who meet the criteria for access to confidential data.*<br><br>*The data underlying the results presented in the study are available from (include the name of the third party*	All relevant data are within the manuscript and its Supporting Information files.

and contact information or URL).<br>• This text is appropriate if the data are owned by a third party and authors do not have permission to share the data.<br><br>* typeset	
Additional data availability information:	Tick here if the URLs/accession numbers/DOIs will be available only after acceptance of the manuscript for publication so that we can ensure their inclusion before publication.

Powered by Editorial Manager® and ProduXion Manager® from Aries Systems Corporation

Manuscript Click here to access/download;Manuscript;Manuscript.docx

Full title: *In-vitro* evaluation of probiotic potential of gut microbes isolated from retail chicken.

Short title: *In-vitro* evaluation of chicken gut microbes for probiotic potential.

Sangram Biswas[1], Lutfor Rahman[2], Susmita Chowdhury[3], Fahmida Khatun[1], Azimun Nahar[4] and Sabina Yasmin[1]*

[1]Department of Biotechnology, Bangladesh Agricultural University (BAU), Mymensingh-2202, Bangladesh.

[2]Department of Microbiology and Hygiene, Bangladesh Agricultural University (BAU), Mymensingh-2202, Bangladesh.

[3]Department of Dietetics and Nutrition, Florida International University (FIU), FL 33199, USA.

[4]Department of Medicine, Bangladesh Agricultural University (BAU), Mymensingh-2202, Bangladesh.

*Corresponding author's email: sabina.yasmin@bau.edu.bd

Abstract

Probiotics are live, non-pathogenic microorganisms that help to improve the host's gut health when administrated in sufficient proportions and are now serving as effective alternatives to antibiotics for managing animal infections and enhancing production. The objective of this study was to isolate, identify and characterize lactic acid bacteria (LAB) strains with excellent probiotic properties from the gastrointestinal tract (GIT) of retail broiler chickens. Samples were enriched in MRS broth at 37°C and plated on MRS agar to isolate distinct colonies of potential probiotic candidates. The isolates underwent a series of standard morphological and biochemical analysis to fulfill the criteria for probiotic characteristics. These analyses included Gram staining, catalase testing, hemolytic activity assays, tolerance assays to NaCl, low pH and bile salts, antagonistic activity assays, antibiotic susceptibility testing, and genotypic identification through 16S rRNA gene sequencing. A total of 40 microbial strains were isolated from the GIT of 20 retail broiler chickens. Of these, 4 LAB strains showed the best probiotic results and were genotypically identified as *Enterococcus faecium* MCI7, *Pedicoccus pentosaceus* MCI10, *Pediococcus pentosaceus* MCC6 and *Pediococcus pentosaceus* MCC12. The selected strains exhibited non-hemolytic activity and demonstrated excellent survival in an acidic environment with a pH level of 3. Furthermore, the strains displayed bile salt ~~suppression~~ in the presence of 0.3% bile salt for 4 hours, ranging from 21.91 to 32.77% and a wide range of antimicrobial activities against various pathogenic bacterial strains with inhibition zones ranging from 10 to 16.5 mm. Moreover, *P. pentosaceus* MCI10, *P. pentosaceus* MCC6 and *P. pentosaceus* MCC12 were sensitive to the majority of the tested antibiotics. Our study identified four LAB strains as promising probiotic candidates for poultry feed additives to effectively establish intestinal microflora, enhance meat quality and growth, and control pathogens.

Keywords: lactic acid bacteria, antagonistic activity, bile salts, GIT, hemolysis.

1.Introduction

The poultry industry significantly contributes to economic growth through job creation and trade, and helps to meet global nutritional needs by providing accessible protein and essential micronutrients [1]. The Food and Agriculture Organization (FAO) and Economic Co-operation and Development (OECD) expect that consumption of poultry meat globally will increase more than 16% and reach 160 million metric tons by 2033 [2]. The global population is projected to reach 9.7 billion by 2050, with global meat consumption expected to increase by 80% due to rising demand for meat protein [3]. In Bangladesh, the annual poultry consumption per capita in 2025 is expected to reach approximately 1.44 kilograms [4]. The poultry production significantly contributes to the national economy of Bangladesh, involving around 1 million entrepreneurs and employing 8 million people [5]. Additionally, this sector has significantly contributed to the nation's advancement of the Sustainable Development Goals (SDGs) by addressing malnutrition and promoting better health by providing protein-rich foods [6]. Therefore, safeguarding the growth of the poultry industry is thought to be crucial to improving the nutritional status and economic development of the Bangladeshi people.

However, the biggest challenges for poultry farming are lack of experience in managing farms, inadequate management practices, insufficient disease control programs, and improper vaccination protocols [7]. Small-scale poultry farming in Bangladesh poses significant public and poultry health risks due to close human-poultry contact and a lack of biosecurity measures [5]. Additionally, farmers use antibiotics to make them grow faster and prevent diseases [8]. The therapeutic use of antibiotics in poultry feed raises significant concerns about the presence of antibiotic residues in poultry products, which can pose risks to consumers [9].

Antimicrobial resistance (AMR) is a natural and ancient phenomenon in bacteria, but human activities, such as the uncontrolled use of antimicrobial agents, have accelerated its emergence and spread. The food animal system becomes a repository for antibiotic-resistant genes as a result of the overuse of

antibiotics, which directly selects resistant microorganisms [10, 11]. Therefore, many European Union (EU) countries banned the marketing and use of antimicrobial growth promoters (AGPs) in feed nutrition since 2006 to their harmful effects, including the development of antibiotic resistance, damaging the natural gut flora, and increased risk of infections [12]. Following the ban on antimicrobial growth promoters (AGPs) and increasing consumer pressure, food animal producers and scientists explore alternatives like probiotics, prebiotics, organic acids, essential oils, phytochemicals, in-feed enzymes and so on [13, 14]. Among these alternatives, probiotics are often preferred due to their ability to directly introduce live beneficial bacteria that can immediately colonize the gut and provide measurable health benefits to maintain productivity and health in livestock, whereas the other compounds primarily act as indirect modulators or substrates, which may not guarantee the same consistent therapeutic outcomes [15]. Probiotics are defined as non-pathogenic live microorganisms which improve host health when administered in adequate amounts [16]. Lactic acid bacteria (LAB) including different species and strains of *Lactobacillus, Bifidobacterium, Bacillus, Lactococcus, Saccharomyces, Streptococcus, Enterococcus, Pedicoccus, Bacteroids* are commonly used potential probiotic microorganisms based on selection criteria [17, 18]. Several previous reports have shown that LAB improves overall flock health and reduces the risk of enteric diseases, boosts growth, enhances immune function, and improves the efficiency of nutrient utilization in poultry [19, 20]. The addition of LAB probiotics as a food supplement has been shown to mitigate heat stress-related diseases in chickens, enhance antibody production, and improve immunity through the modulation of Toll-like receptor (TLR) signaling [21, 22]. Furthermore, Metabolic components derived from probiotics, including bacteriocins, amines and hydrogen peroxide which have the ability to interact with particular targets within various metabolic pathways to enhance cell proliferation through the inhibition of regular apoptosis and the stimulation of cell differentiation [23]. It has been reported that adding probiotic supplements to broilers at a dosage of 0.5 g/kg improves the pH and texture of the meat [24].

Currently, there's a rising concern about the heavy use of antibiotics in poultry production, especially in developing countries like Bangladesh, leading to a growing demand for poultry probiotics [25]. However, the limited stability and variable performance of probiotic strains often hinder their widespread use [26]. Probiotic strains that are derived from their natural hosts tend to be more compatible with the gastrointestinal tract, allowing for better adherence, survivability, and efficacy than strains from other sources. Thus, the host-specific probiotic strains development is essential for maximizing health benefits and improving livestock production performance [27]. Therefore, the objective of this study is to isolate, identify and characterize LAB strains with excellent probiotic properties from the gastrointestinal tract (GIT) of retail broiler chickens for potential integration into poultry farming practices.

2. Materials and Methods

2.1 Isolation and culturing of probiotics

The gastrointestinal tracts (intestines and cecum) of twenty broiler chickens were collected from different retail poultry shops in Mymensingh, Bangladesh (Table 1). The samples were maintained on ice during transportation to the laboratory and were promptly washed using 0.9% sodium chloride (NaCl) solution upon arrival. After washing each section of GIT, a 5 g sample from each section was transferred to 20 ml of de Man, Rogosa, and Sharpe (MRS) broth (Hi-Media, India) for enrichment and incubated aerobically at 37°C for 24 hours. Cultures displaying visible growth were selected, streaked onto MRS agar plates, and incubated anaerobically at 37°C for 24–72 hours. Only colonies showing greyish and creamy white appearance (presumptive for LAB) were selected and undergo three consecutive transfers on MRS agar plates to purify individual colonies [28, 29].

Table 1. Location details of the GIT samples collection, along with the number of isolates that were assessed for their probiotic characteristics.

SL No.	Location	Number of Chicken Collected	Bacterial Isolate No.	Coordinates
1	K.R. MARKET, BAU	4	MCI1, MCI2, MCI3, MCI4<br>MCC1, MCC2, MCC3, MCC4	24.72693,<br>90.43662
2	Kewatkhali Bazar	4	MCI5, MCI6, MCI7, MCI8<br>MCC5, MCC6, MCC7, MCC8	24.73830,<br>90.42520
3	Mechua Bazar	4	MCI9, MCI10, MCI11, MCI12<br>MCC9, MCC10, MCC11, MCC12	24.738506,<br>90.424664
4	Shes More Bazar	4	MCI13, MCI14, MCI15, MCI16<br>MCC13, MCC14, MCC15, MCC16	24.71771,<br>90.44414
5	Sutiakhali Bazar	4	MCI17, MCI18, MCI19, MCI20<br>MCC17, MCC18, MCC19, MCC20	24.697372<br>90.454762

Note: MCI-Mymensingh Chicken Intestine, MCC- Mymensingh Chicken Cecum

2.2 Morphological and Biochemical Characterization

Pure cultures were characterized morphologically using Gram staining and analyzed biochemically with catalase tests and carbohydrate fermentation profiles according to standard procedures [30].

Potential probiotic strains were identified as isolates displaying Gram-positive cocci morphology with negative catalase activity and were preserved at -20°C in MRS broth containing 28% (w/v) glycerol.

2.3 Assays of Probiotics

2.3.1 Hemolytic Activity

Fresh bacterial cultures were streaked onto Columbia blood agar base (HiMedia India) plates supplemented with 5% (v/v) sheep blood. Following incubation at 37°C for 24 hours, the isolates were assessed for clear zones around the colonies. Clear, colorless/lightened yellow zones depicting total Red Blood Cell (RBC) lysis are considered as beta (β) hemolysis. Alpha (α) hemolysis is characterized by a small greenish zone around the colonies due to the conversion of hemoglobin to methemoglobin, and gamma (γ) hemolysis refers to the absence of any hemolytic zones, indicating no hemolysis. Colonies displaying non hemolytic activity were selected, and those displaying beta or alpha hemolysis pattern were excluded [31].

2.3.2 NaCl tolerance assay

Salt tolerance of the bacterial strains was assessed by inoculating 50 µL of overnight-grown culture (1% v/v) into 5 mL of MRS broth supplemented with 2.0%, 4.0%, and 6.0% (w/v) NaCl (Sodium Chloride; Merck, India). After incubating for 24 hours at 37°C, bacterial growth was determined by measuring the optical density at 600 nm (OD^{600}) [32].

2.3.3 Survival Under Low pH

The acid resistance assay was conducted using the protocol described by Amelia et al. (2020) [33] with minor modifications. Briefly, the MRS broth medium was prepared at pH of 3 by using 1N HCl. The bacterial isolates were first cultured overnight in MRS broth under anaerobic conditions at 37°C. Then, 1 ml overnight bacterial culture was inoculated in 9ml broth medium and incubated at 37°C for 3 h. Samples were collected at the beginning (0 h) and after 3 hours of incubation. These samples were serially diluted tenfold in sterile saline solution (5.8 g/L NaCl), and 1 mL of each dilution was plated

on MRS agar. Plates were incubated aerobically at 37°C for 48 to 72 hours. The survival rate was measured by counting colony-forming units at both 0 and 3 hours, following the method described by Bazireh et al. (2020) [34].

2.3.4 Tolerance to Bile Salt

Bile salt tolerance was assessed using a slightly modified version of the method previously described by Naim et al. (2019) [35]. In brief, overnight bacterial cultures were inoculated (1% v/v) into two media: MRS broth containing 0.3% (w/v) ox-bile salts and regular MRS broth without bile salts as a control. Both samples were incubated at 37°C, and the optical density (OD^{600}) was measured using a spectrophotometer at 0 h and 4 h after incubation. Subsequently, the percentage of growth suppression was measured by using the following formula:

$$\text{\% of suppression} = \frac{\text{Growth in control} - \text{Growth in bile salt}}{\text{Growth in control}} \times 100$$

2.3.5 Antimicrobial Activity

Antimicrobial activity assays were carried out using the well diffusion method with pathogenic bacteria *Escherichia coli*, Methicillin-resistant *Staphylococcus aureus* (MRSA), *Enterococcus faecalis*, *Salmonella Typhimurium* and *Klebsiella pneumoniae*. The LAB isolates were grown overnight in MRS broth at 37°C, and the selected pathogens were cultured under the same conditions in Buffered Peptone Water (BPW). These pathogenic organisms were spread on Mueller-Hinton agar and the wells were cut on plates. Then, cell-free supernatants (CFS) were prepared by centrifugation of LAB cultures at 6000 rpm for 10 minutes and 100 µL aliquots were deposited into each well. After 24 h of incubation at 37^0C, antimicrobial activity of the putative probiotic candidates was determined by measuring the diameter of clear zones of inhibition around the wells using a transparent ruler [35].

2.3.6 Antibiotic Sensitivity

The antibiotic resistance profile of LAB isolates was assessed on MRS agar using the antibiotic disc diffusion method. The agar medium was poured into plates and allowed to solidify at room temperature. The LAB bacterial cultures actively growing overnight, were uniformly distributed across the solidified agar surface using a sterile spreader and allowed to dry. Once the cultures were evenly spread, 14 antibiotic disks were placed on the surface of the agar plate. Subsequently, the plates were incubated at 37°C for 24 hours. The antibiotic susceptibility pattern of the isolates was assessed using nalidixic acid (30 µg/disc), ciprofloxacin (5 µg/disc), chloramphenicol (30 µg/disc), cefepime (30 µg/disc), imipenem (10 µg/disc), streptomycin 300, gentamicin (10 µg/disc), ampicillin (10 µg/disc), cefoxitin (30 µg/disc), sulfamethoxazole (25 µg/disc), cefotaxime (30 µg/disc), oxytetracycline (30 µg/disc), ceftazidime (30 µg/disc), and azithromycin (30 µg/disc). Following incubation, the diameter of inhibition zones around each disk was measured using a transparent ruler. Based on the measured diameters, inhibition zones were categorized as susceptible (≥21 mm), intermediate (16–20 mm), or resistant (≤15 mm) for each antibiotic [36].

2.4 Molecular Identification by 16S rRNA sequencing

The LAB strains that fulfilled the probiotic selection criteria underwent molecular identification via 16S rRNA gene sequencing. DNA of four isolates was extracted using TIANamp Bacteria DNA Kit (TIANGEN), followed by PCR amplification in 35 µl reactions containing 16 µl of OneTaq Quick-Load 2X Master Mix, 1 µl each of forward (27F: 5'-AGAGTTTGATCMTGGCTCAG-3') and reverse primers (1492R: 5'-TACGGYTACCTTGTTACGACTT-3') [29], 14 µl of DNase-free water, and 3 µl of DNA template. The thermal cycling protocol (BIORAD, United States) included initial denaturation at 95°C for 5 min, followed by 35 amplification cycles (94°C for 1 min, 60°C for 1.30 min, and 72°C for 2 min), with a final extension at 72°C for 7 min. After amplification, 5 µl of PCR products were analyzed by 1.0% (w/v) agarose gel electrophoresis and visualized under UV transillumination using ImageMaster (BIORAD, United States). PCR products of the expected size (1.5 kb) were purified and subjected to Sanger sequencing. The LAB strains were identified by sequence homology searches

using the basic local alignment search tool (BLAST) against GenBank reference database. Thereafter, the sequences were registered in the NCBI (https://www.ncbi.nlm.nih.gov/genbank/) database and assigned with accession numbers. To analyze evolutionary relationships among isolates, phylogenetic trees were generated using MEGA version 12 [37], employing neighbor-joining statistical method, maximum likelihood substitution, and 1,000-repeat Bootstrap analysis [38].

2.5 Statistical Analysis

Statistical analyses were conducted using SPSS version 26.0 (SPSS, Chicago, IL, United States), Microsoft Excel, and GraphPad Prism 8.0.2 (GraphPad Software, San Diego, CA, USA). Mean differences between treatments were analyzed using one-way ANOVA with significance determined at $P < 0.05$. All experiments data were performed triplicate and was expressed as means ± standard deviations (SD).

3. Results

3.1 Characterization and Identification of Isolates

In total, 40 bacterial colonies (with 20 each from intestine and cecum) were randomly selected from the cultures of samples taken from the GIT of the 20 chickens. The initial 11 probable probiotic bacterial candidates were identified among 40 bacterial colonies based on their typical morphological appearance (5 small pinpointed, grayish white colonies and 6 small pinpointed, creamy white colonies), Gram-positive, catalase-negative, and cocci-shaped characteristics. Further identification through sugar fermentation profiling indicated that the isolates likely belonged to be *Enterococcus* and *Pedicoccus* species (S1 Fig and Table 2).

Table 2. Morphological and biochemical characteristics of selected isolates.

Strains	Cell Morphology	Gram	Catalase	Glucose	Sucrose	Lactose	Maltose	Fructose	D-Mannitol
MCI2	Small pinpointed, Grayish white and Cocci	+	-	+	-	+	+	+	-
MCI6	Small pinpointed, Grayish white and Cocci	+	-	+	+	+	+	+	-
MCI7	Small pinpointed, Grayish white and Cocci	+	-	+	+	+	+	+	+
MCI9	Small pinpointed, Creamy white and Cocci	+	-	+	-	+	+	+	-
MCI10	Small pinpointed, Creamy white and Cocci	+	-	+	+	+	+	+	+
MCI11	Small pinpointed, Creamy white and Cocci	+	-	+	+	+	+	+	+
MCC5	Small pinpointed, Grayish white and Cocci	+	-	+	+	+	+	+	+
MCC6	Small pinpointed, Creamy white and Cocci	+	-	+	+	+	+	+	+
MCC10	Small pinpointed, Creamy white and Cocci	+	-	+	+	+	+	+	+

MCC12	Small pinpointed, Creamy white and Cocci	+	-	+	+	+	+	+	+
MCC15	Small pinpointed, Grayish white and Cocci	+	-	+	-	+	+	+	-

3.2 Assays of Probiotics

3.2.1 Hemolytic ability

In terms of hemolytic activity, seven of the eleven isolates (MCI2, MCI7, MCI10, MCI11, MCC6, MCC10 and MCC12,) showed no change in the color of the media, indicating non-hemolytic activity (γ-hemolysis) (S1 Table). No zones appeared around the colonies (S2 Fig), confirming the absence of RBC lysis and indicating that they are considered safe and best. They were used for subsequent assays.

3.2.2 NaCl tolerance

All the isolates demonstrated the ability to grow at NaCl concentrations of 2%, 4%, and 6%, with a general decrease in OD^{600} as NaCl concentration increased (Fig. 1). At 2%, 4%, and 6% NaCl, isolate MCC6 displayed the highest salt tolerance with OD^{600} values of 1.688 ± 0.04, 1.539 ± 0.03, and 1.422 ± 0.04, respectively, while isolate MCI11 consistently exhibited the lowest OD^{600} across all concentrations. For the higher concentrations of 6% NaCl, very weak growth was observed for MCI2 and MCI11, with OD^{600} values 0.123 ± 0.02 and 0.078 ± 0.01 respectively, and they were excluded from further analysis due to their poor performance. Statistical analysis confirmed significant differences in salt tolerance among isolates ($p < 0.05$).

Fig 1. Characterization of the selected isolates using NaCl tolerance assay.

3.2.3 Survival Under Low pH

After analysis of NaCl tolerance, selected 5 isolates were examined to pH 3. The colony counts of potential acid-tolerant isolates were determined on MRS agar at pH 3 following 0 and 3 hours of incubation. All isolates were able to survive at pH 3.0 after 3h incubation except MCC10. Strains MCI7, MCI10, MCC6 and MCC12 showed the relatively highest survival rate ranging from (87.29 to 89.89%) after 90 min of incubation, while MCC10 showed no viability excluded for further study. The results are shown in Table 3.

Table 3. Survivability and acid tolerance of selected isolates in acidic conditions

Isolates	pH tolerance (log CFU/ml)		Survival rate (%)
	0 h	3 h	
MCI7	8.18 ± 0.13	7.14 ± 0.1	87.29
MCI10	8.01 ± 0.15	7.13 ± 0.1	89.01
MCC6	7.88 ± 0.08	7.02 ± 0.08	89.09
MCC10	7.09 ± 0.01	ND	ND
MCC12	8.11 ± 0.01	7.29 ± 0.07	89.89

Note: ND - not determined; ± indicates standard deviation from the mean. Values are significantly different ($P < 0.05$) at 5% level of probability.

3.2.4 Bile Salt Tolerance

After 4 h incubation at 37°C for the assessment of bile salt tolerance, isolates MCI10 and MCC6 showed the highest resistance, with growth inhibition capabilities 25.99 ± 0.02% and 21.91 ± 0.01% respectively. However, isolate MCI7 exhibited the most suppression 32.78 ± 0.02 %, while MCC12

demonstrated 27.46 ± 0.01%. In general, all the isolates exhibited bile salt tolerance above 50% (Fig 2). Additionally, MCC6 showed the greatest OD^{600}, i.e., 0.438 ± 0.01 in the presence of bile salts, while MCI7 had the lowest value of 0.255 ± 0.02 (S3 Table).

Fig 2. Effect of bile salts on growth inhibition rates (suppression) of four selected isolates.

3.2.5 Antimicrobial Activity

The antimicrobial activity of the isolated strains was evaluated against five types of pathogens, and the results are shown in Table 4. Using the agar well diffusion assay, selected 4 LAB strains (MCI7, MCI10, MCC6 and MCC12) showed no inhibition activity against *S. aureus* (MRSA), while MCI10 and MCC6 strains were able to inhibit the *E. faecalis*. All strains demonstrated broad-spectrum activity, inhibiting three types of pathogens including *E. coli, S. typhimurium* and *K. pneumoniae*.

Table 4. Antimicrobial activities against five pathogens.

Isolate No.	Diameter (mm) of inhibition zone				
	E. coli	***S. aureus* MRSA**	***E. faecalis***	***S. Typhimurium***	***K. pneumoniae***
MCI7	10	-	-	13	11.5
MCI10	12	-	12	15.5	13
MCC6	14.5	-	11	16.5	14
MCC12	12	-	-	16	13

The zone diameter measurements represent the mean values from two experimental trials, expressed in millimeters (mm).

3.2.6 Evaluation of Antibiotic Susceptibility

The antibiotic susceptibility results of the four selected LAB isolates were tested against different antibiotics. All the isolates exhibited differing levels of antibiotic sensitivity and are presented in Table 5. The results indicated that all the strains were resistant to nalidixic acid, ciprofloxacin, cefoxitin, sulfamethoxazole and tetracycline, while each of the strains was susceptible to ciprofloxacin. No antibiotic resistance patterns were observed for cefepime, imipenem and cefotaxime except MCI7. Three of the four strains were effective inhibitors against gentamicin and azithromycin.

Table 5. Susceptibility of potential LAB probiotic strains against some antibiotics measured by agar disc diffusion.

Strains	NA	CIP	C	FEP	IMP	S	CN	AM	FOX	SXT	CTX	T	CAZ	AZM
MCI7	R	R	S	R	R	R	R	R	R	R	R	R	R	R
MCI10	R	R	S	S	S	I	I	I	R	R	S	R	I	I
MCC6	R	R	S	S	S	S	I	I	R	R	S	R	R	I
MCC12	R	R	S	S	S	S	I	S	R	R	S	R	R	I

R - Resistant; S -Sensitive; and I - Intermediate.

NA - nalidixic acid, CIP - ciprofloxacin, C - chloramphenicol, FEP - cefepime, IMP - imipenem, S - streptomycin, CN - gentamicin, AM - ampicillin, FOX - cefoxitin, SXT - sulfamethoxazole, CTX - cefotaxime, T - tetracycline, CAZ – ceftazidime and AZM - azithromycin.

3.3 Molecular Characterization

Molecular analysis was used to identify potential strains of LAB probiotics. All four LAB isolates displayed a band of 1500 bp in size after electrophoresis of the PCR amplification products on an

agarose gel (Fig 3). Sequences of the 16S rRNA gene were analyzed using BLAST and subsequently deposited in the GenBank database, where accession numbers were assigned (Table 6).

Fig 3. Electrophoretic analysis of amplified 16S rRNA PCR products using agarose gel: lane M, 100bp Marker (ADDBIO, SOUTH KOREA), Lanes 1, 2, 3, and 4 are positive lactic acid bacteria (LAB) strains MCI7, MCI10, MCC6 and MCC12 at 1500 bp.

Table 6. Species-level identification of probiotic LAB isolates using 16s rRNA sequencing

Strains	Sequence Accession Number	Sequence Identity %
Enterococcus faecium strain MCI7	PQ819573	99.15
Pediococcus pentosaceus strain MCI10	PQ819578	98.97
Pediococcus pentosaceus strain MCC6	PQ819574	98.87
Pediococcus pentosaceus strain MCC12	PQ819579	99.16

3.4 Phylogeny analysis

A phylogenetic tree (Fig 4) was constructed using the neighbor-joining method based on evolutionary distances derived from the aligned 16S-rRNA sequences. The phylogenetic tree demonstrated the relationship of potential LAB strains to the isolates and type strains based on sequence differences. The 4 LAB isolates were identified as: *E. faecium* MCI7, *P. pentosaceus* MCI10, *P. pentosaceus* MCC6 and *P. pentosaceus* MCC12. Each species formed distinct groups (clades) with high bootstrap support, confirming their evolutionary separation and close relationship.

Fig 4. Phylogenetic tree constructed from 16S rRNA sequences of 4 LAB isolates and type strains. The sequenced LAB isolates are indicated in bold font.

4. Discussion

In recent years, probiotics have gained considerable attention as promising alternatives to growth-promoting antibiotics in poultry production, while mitigating concerns about antimicrobial resistance and food safety [39]. Most probiotic strains are isolated from the intestinal microbiota of humans and animals, as well as from fermented dairy products [40]. Host specific probiotics development is essential than strains isolated from the other sources because of their enhanced ability to colonize intestinal mucosa, allowing them to integrate more effectively into the host's gut microbiome [29]. Furthermore, direct *in vivo* evaluation of probiotics is often costly and time-consuming. As a result, *in vitro* testing is widely used to identify strains with the most beneficial properties. Additionally, evaluating the health effects of a particular probiotic strain in live animals can be very difficult and expensive [41]. In this study, LAB strains were isolated from healthy broiler chickens and evaluated *in vitro* for their potential as poultry probiotics.

In our study, we successfully isolated and characterized potential LAB from 20 healthy broiler chicken individuals collected from retail chicken shops in Mymensingh (Bangladesh). Out of 40 microbial isolates, 11 were phenotypically and biochemically identified to be probable probiotic candidates. Of these, at least 4 LAB strains were genetically confirmed as *Enterococcus faecium* (MCI7) and *Pediococcus pentosaceus* (MCI10, MCC6 and MCC12) species.

The FAO recommends that microbial strain being considered for probiotic use must be safe to their host [42]. Hemolysis is a virulence factor frequently associated with pathogenic microorganisms that produce hemolysins to break down membrane phospholipids, leading to cell lysis [43]. For safety assurance, the selection of non-hemolytic strains is recommended, as this characteristic indicates an absence of virulence potential in probiotic candidates [44]. In our study, 7 out of 11 isolates showed γ-

hemolytic or no hemolytic activity and were therefore chosen for further evaluation due to their safety as potential probiotics. Many researchers have reported similar results in the literature indicating that probiotics typically do not exhibit any hemolytic activity [33, 45, 46].

Our study revealed that the selected LAB isolates showed excellent tolerance to 2, 4 and 6% NaCl, which may be due to the accumulation of compatible solutes and expression of specific stress proteins that collectively enhance their resilience to osmotic stress [47]. Similarly, Reuben et al., (2019) [29] found that all isolates of lactic acid bacteria from GIT of chicken could survive at a concentration of 6.5% NaCl, and weak growth was reported at 10% NaCl. An earlier study showed that LAB isolated from milk and dairy products, meats, chicken faeces and other sources can survive in NaCl concentrations ranging from 1.0% to 9.0% [48, 49]. These results underscore the promising characteristics of potential LAB strains as probiotics, such as promoting bacterial growth and producing valuable metabolites, with significant implications for food preservation and production.

The selection of good probiotic bacteria depends on their ability to survive low pH conditions, pass through the digestive tract, and successfully colonize the host's gut [50]. In our study, isolates MCI7, MCI10, MCC6 and MCC12 displayed high survival rates ranging from 87.29% to 89.89% at pH 3.0, indicating their potential to survive the harsh acidic conditions of the host gut. Similarly, isolates SA 135, SA 151, and ST 80 isolated from human salivary and fecal sources showed high survival rates ranging from 97.4% to 99.32% under acidic conditions at pH 3.0 [34]. Park et al., (2023) reported that survival rates of *Pediococcus pentosaceus* CACC616 strains isolated from fecal samples of pigs was 88.4% at pH 2.5 for 2 hours incubation [51]. The findings of our study indicate that the ability of our potential LAB probiotic strains to survive in low pH conditions is strain-specific.

Bile, a digestive fluid produced in the liver and stored in the gallbladder, can be toxic to certain microorganisms. To effectively colonize the gut, probiotics must be able to withstand and survive in the presence of bile [52]. The concentrations of bile salts in the duodenum and cecum of the chicken

gastrointestinal tract are approximately 0.175% and 0.008% respectively [53]. However, numerous studies have taken into account the average intestinal bile concentration is 0.3% (w/v) in order to evaluate the bile salt tolerance of probiotic LAB strains. [54]. In our study, all the selected LAB strains demonstrated moderate to good bile salt tolerance under 0.3% bile salt conditions after 4h incubation (S3 Table), indicating their ability to survive and grow in the gastrointestinal environment. Similarly, Shin et al., (2008) and Shokryazdan et al., (2014) [54, 55], reported good tolerance to 0.3% bile salts by all LAB strains isolated from chicken.

Zoonotic diseases and foodborne bacterial pathogens can cause high mortality and morbidity in poultry, leading to substantial economic losses in the poultry industry [56]. One of the important criteria for selecting a good probiotic candidate is its ability to effectively inhibit these pathogenic bacteria [57]. LAB exhibits antagonistic activity through the secretion of various antimicrobial substances, including bacteriocins, organic acids such as hydrogen peroxide, lactic acid, diacetyl, and carbon dioxide [58]. In our study, the selected strains exhibited antagonistic activity against *E. coli, S. Typhimurium* and *K. pneumoniae* while no antimicrobial activity was shown against *S. aureus* (MRSA). However, the inhibitory effects of MCI10 and MCI6 were found against *E. faecalis* (Table 4). Our findings align with numerous earlier studies showing that LAB isolated from poultry display a broad range of antagonistic effects against different pathogens [29, 55, 59]. In contrast, Kizerwetter-Swida and Binek [60] found that LAB from chicken intestinal tracts more effectively inhibit Gram-positive pathogens (including *Staphylococcus aureus* and *Clostridium perfringens*) compared to Gram-negative pathogens (including *Salmonella* and *E. coli*). However, de Almeida Júnior et al., (2015) [61] found that the inhibitory activity of LAB showed no relationship with whether the tested pathogens were Gram-positive or Gram-negative. Spanggaard et al., (2001) [62] previously observed that probiotics can inhibit the establishment of heterochthonous bacteria in the gastrointestinal tract (GIT) by managing pathogens, indicating that probiotics could play a crucial role in pathogen control.

As a key feature, probiotic microbial strains should not possess or acquire any antibiotic-resistance genes, which could be transferred to enteric pathogens [63]. Therefore, the assessment of the antimicrobial susceptibility of isolated bacteria is crucial for selecting potential probiotic strains. The antibiotic sensitivity test clarified that isolates MCI10, MCC6 and MCC12 were resistant to cefoxitin, ciprofloxacin, nalidixic acid, sulfamethoxazole and tetracycline (Table 5). Reuben et al.,[29] reported that LAB strains isolated from poultry gastrointestinal tract were resistant to ciprofloxacin and tetracycline. Also, several studies have evidenced the resistance to various antibiotics by LAB [51, 64, 65]. Isolate MCI7 was resistant to all tested antibiotics except chloramphenicol. A possible explanation for this result may be the horizontal transmission of antibiotic-resistant genes [66] and the overuse of antibiotics in Bangladesh.

5. Conclusions

This research identified four potential probiotic bacteria from the gastrointestinal tracts of retail broiler chickens. The *in vitro* results indicated that, in particular, LAB strains *P. pentosaceus* MCI10, *P. pentosaceus* MCC6 and *P. pentosaceus* MCC12 showed relatively good antipathogenic activity, antibiotic sensitivity, low pH and bile salt tolerance, therefore, they could have the greatest potential for probiotic use in chickens as feed additives to combat pathogens, improving poultry health, enhancing productivity, and reducing antibiotic reliance. Further investigation is necessary to evaluate the health benefits and protective effects of these new probiotic candidates *in vivo*.

Supporting information

S1 Fig. Morphology and Gram stain results of four selected LAB probiotic strains: MCI7, MCI10, MCC6 and MCC12. (A) Morphology results of MCI7; (B) Gram stain results of MCI7; (C) Morphology results of MCI10; (D) Gram stain results of MCI10; (E) Morphology results of MCC6; (F) Gram stain results of MCC6; (G) Morphology results of MCC12; and (H) Gram stain results of MCC12.

S2 Fig. Results of the hemolytic activity of the 4 selected lactic acid bacteria (LAB) probiotic strains from chicken GIT. A) MCI7 B) MCI10 C) MCC6 and D) MCC12.

S3 Fig. The inhibitory effects of isolated probiotic strains against pathogenic indicator bacteria. The pathogenic indicator bacteria were (A) *E. coli*; (B) *S. aureus*; (C) *E. faecalis*; (D) *S. typhimurium*; and (E) *K. pneumonia*. In each MHA agar plate, (a) was added to the MRS broth as control; (b) was added to the cell-free supernatant of MCI7; (c) was added to the cell-free supernatant of MCI10; (d) was added to the cell-free supernatant of MCC6 and (e) was added to the cell-free supernatant of MCC12.

S4 Fig. Antibiotic susceptibility pattern of isolated probiotic strains against different antibiotics was determined by disk diffusion assay. A) & B) *Enterococcus faecium* strain MCI7; C) & D) *Pediococcus pentosaceus* strain MCI10; E) & F) *Pediococcus pentosaceus* strain MCC6 and G) & H) *Pediococcus pentosaceus* strain MCC12.

S1 Table. Hemolytic activities of potential lactic acid bacteria (LAB) probiotic strains from chicken GIT.

S2 Table. Survival of potential lactic acid bacteria (LAB) probiotic strains at 2, 4, and 6% NaCl concentration estimated through measuring OD^{600}.

S3 Table. Bile salt resistance of potential lactic acid bacteria (LAB) probiotic strains, estimated by measuring OD^{600} in the presence and absence of bile salts.

Acknowledgments

We acknowledge the staff of the Biotechnology Laboratory and the Zoonotic Disease Laboratory in Bangladesh Agricultural University for their logistical, and operational support throughout the study. We also express our gratitude to the members of the local community from whom we collected our samples.

Author contribution

Conceptualization: Sangram Biswas, Sabina Yasmin, Azimun Nahar.

Data curation: Sangram Biswas, Lutfor Rahman.

Formal analysis: Sangram Biswas.

Investigation: Sabina Yasmin, Azimun Nahar, Fahmida Khatun.

Methodology: Sangram Biswas, Lutfor Rahman.

Supervision: Sabina Yasmin, Azimun Nahar, Fahmida Khatun.

Validation: Sabina Yasmin, Azimun Nahar, Fahmida Khatun**.**

Visualization: Sangram Biswas, Lutfor Rahman.

Writing – original draft: Sangram Biswas.

Writing – review & editing: Sangram Biswas, Lutfor Rahman, Susmita Chowdhury, Fahmida Khatun, Azimun Nahar, Sabina Yasmin.

Institutional Review Board Statement: The animal study protocol was approved by the Animal Welfare and Experimentation Ethics Committee, Bangladesh Agricultural University, Mymensingh-2202, Bangladesh (AWEEC/BAU/2025(2)/32(a), Date: 25-05-2025).

Competing interests: The authors have declared that no competing interests exist.

References

1. Mottet A, and Tempio G. Global poultry production: current state and future outlook and challenges. World's Poultry Science Journal. 2017;73(2):245-56. doi: https://doi.org/10.1017/S0043933917000071.

2. Clements M. Ever-growing demand for poultry shows no signs of abating.: WATTPoultry; 2024. Available from: https://www.wattagnet.com/blogs/poultry-around-the-world/blog/15679810/evergrowing-demand-for-poultry-shows-no-signs-of-abating.

3. Nadathur S, Wanasundara J, Scanlin L. Proteins in the diet: Challenges in feeding the global population. Sustainable protein sources: Elsevier; 2017. p. 1-19.

4. Department SR. Poultry consumption per capita in Bangladesh from 2006 to 2025.: Statista; 2024. Available from: https://www.statista.com/statistics/757647/bangladesh-poultry-consumption-per-capita/.

5. Hub OHP. Poultry in Bangladesh: UKRI GCRF One Health Poultry Hub; 2024. Available from: https://www.onehealthpoultry.org/where-we-work/bangladesh/poultry-in-bangladesh/.

6. Vlaicu PA, Untea AE, Oancea AG. Sustainable poultry feeding strategies for achieving zero hunger and enhancing food quality. Agriculture. 2024;14(10):1811. doi: https://doi.org/10.3390/agriculture14101811.

7. Zaman MH, Zaman M, Rakib AA, Bhuiyan MMI. The Poultry Industry of Bangladesh: An Overview. London: SAGE Publications: SAGE Business Cases Originals; 2021. Available from: https://sk.sagepub.com/cases/the-poultry-industry-of-bangladesh-an-overview.

8. Sardar D, Afsana S, Habib A, Hossain T. Dietary effects of multi-strain probiotics as an alternative to antibiotics on growth performance, carcass characteristics, blood profiling and meat quality of broilers. Veterinary Integrative Sciences. 2024;23(2):1-17. doi: https://doi.org/10.12982/VIS.2025.059.

9. Muhammad J, Khan S, Su JQ, Hesham AE-L, Ditta A, Nawab J, et al. Antibiotics in poultry manure and their associated health issues: a systematic review. Journal of Soils and Sediments. 2020;20:486-97. doi: https://doi.org/10.1007/s11368-019-02360-0.

10. Gao P, Ma C, Sun Z, Wang L, Huang S, Su X, et al. Feed-additive probiotics accelerate yet antibiotics delay intestinal microbiota maturation in broiler chicken. Microbiome. 2017;5(1):91. Epub 2017/08/05. doi: https://doi.org/10.1186/s40168-017-0315-1. PMID: 28768551.

11. Hossain MT, Sardar D, Afsana S, Datta M, Habib MA. Comparative analysis between multi-strain probiotics and antibiotic as starter feed supplement of poultry on growth performance, serum metabolites and meat quality. Vet Anim Sci. 2024;24:100346. Epub 2024/06/19. doi: https://doi.org/10.1016/j.vas.2024.100346. PMID: 38895706; PubMed Central PMCID: PMC11184486.

12. Anadón A. WS14 The EU ban of antibiotics as feed additives (2006): alternatives and consumer safety. Journal of Veterinary Pharmacology and Therapeutics. 2006;29:41-4. doi: https://doi.org/10.1111/j.1365-2885.2006.00775_2.x.

13. Arsène MMJ, Davares AKL, Andreevna SL, Vladimirovich EA, Carime BZ, Marouf R, et al. The use of probiotics in animal feeding for safe production and as potential alternatives to antibiotics. Vet World. 2021;14(2):319-28. Epub 2021/03/30. doi: https://doi.org/10.14202/vetworld.2021.319-328. PMID: 33776297; PubMed Central PMCID: PMC7994123.

14. Mak PHW, Rehman MA, Kiarie EG, Topp E, Diarra MS. Production systems and important antimicrobial resistant-pathogenic bacteria in poultry: a review. J Anim Sci Biotechnol. 2022;13(1):148. Epub 2022/12/14. doi: https://doi.org/10.1186/s40104-022-00786-0. PMID: 36514172; PubMed Central PMCID: PMC9749270.

15. Hill C, Guarner F, Reid G, Gibson GR, Merenstein DJ, Pot B, et al. Expert consensus document. The International Scientific Association for Probiotics and Prebiotics consensus statement on the scope and appropriate use of the term probiotic. Nat Rev Gastroenterol Hepatol. 2014;11(8):506-14. Epub 2014/06/11. doi: https://doi.org/10.1038/nrgastro.2014.66. PMID: 24912386.

16. Reid G, Gadir AA, Dhir R. Probiotics: Reiterating What They Are and What They Are Not. Front Microbiol. 2019;10:424. Epub 2019/04/02. doi: https://doi.org/10.3389/fmicb.2019.00424. PMID: 30930863.

17. Mishra S, Acharya S. A brief overview on probiotics: the health friendly microbes. Biomedical and Pharmacology Journal. 2021;14(4):1869-80. doi: https://dx.doi.org/10.13005/bpj/2285.

18. Mountzouris KC, Tsirtsikos P, Kalamara E, Nitsch S, Schatzmayr G, Fegeros K. Evaluation of the efficacy of a probiotic containing Lactobacillus, Bifidobacterium, Enterococcus, and Pediococcus strains in promoting broiler performance and modulating cecal microflora composition and metabolic activities. Poult Sci. 2007;86(2):309-17. Epub 2007/01/20. doi: https://doi.org/10.1093/ps/86.2.309. PMID: 17234844.

19. Abd El-Hack ME, El-Saadony MT, Shafi ME, Qattan SYA, Batiha GE, Khafaga AF, et al. Probiotics in poultry feed: A comprehensive review. J Anim Physiol Anim Nutr (Berl). 2020;104(6):1835-50. Epub 2020/10/01. doi: https://doi.org/10.1111/jpn.13454. PMID: 32996177.

20. Lutful Kabir SM. The role of probiotics in the poultry industry. Int J Mol Sci. 2009;10(8):3531-46. Epub 2010/01/30. doi: https://doi.org/10.3390/ijms10083531. PMID: 20111681; PubMed Central PMCID: PMC2812824.

21. Asgari F, Falak R, Teimourian S, Pourakbari B, Ebrahimnezhad S, Shekarabi M. Effects of Oral Probiotic Feeding on Toll-Like Receptor Gene Expression of the Chicken's Cecal Tonsil. Rep Biochem Mol Biol. 2018;6(2):151-7. Epub 2018/05/17. PMID: 29765997; PubMed Central PMCID: PMC5941120.

22. Zulkifli I, Abdulllah N, Azrin NM, Ho YW. Growth performance and immune response of two commercial broiler strains fed diets containing Lactobacillus cultures and oxytetracycline under heat stress conditions. Br Poult Sci. 2000;41(5):593-7. Epub 2001/02/24. doi: https://doi.org/10.1080/713654979. PMID: 11201439.

23. Plaza-Diaz J, Ruiz-Ojeda FJ, Gil-Campos M, Gil A. Mechanisms of action of probiotics. Advances in nutrition. 2019;10:S49-S66. doi: https://doi.org/10.1093/advances/nmy063.

24. Mohammed AA, Zaki RS, Negm EA, Mahmoud MA, Cheng HW. Effects of dietary supplementation of a probiotic (Bacillus subtilis) on bone mass and meat quality of broiler chickens. Poult Sci. 2021;100(3):100906. Epub 2021/02/02. doi: https://doi.org/10.1016/j.psj.2020.11.073. PMID: 33518351; PubMed Central PMCID: PMC7936156.

25. Abou-Jaoudeh C, Andary J, Abou-Khalil R. Antibiotic residues in poultry products and bacterial resistance: A review in developing countries. J Infect Public Health. 2024;17(12):102592. Epub 2024/11/18. doi: https://doi.org/10.1016/j.jiph.2024.102592. PMID: 39551017.

26. Li X, Li W, Zhao L, Li Y, He W, Ding K, et al. Characterization and Assessment of Native Lactic Acid Bacteria from Broiler Intestines for Potential Probiotic Properties. Microorganisms. 2024;12(4). Epub 2024/04/27. doi: https://doi.org/10.3390/microorganisms12040749. PMID: 38674693; PubMed Central PMCID: PMC11052334.

27. Dowarah R, Verma AK, Agarwal N, Singh P, Singh BR. Selection and characterization of probiotic lactic acid bacteria and its impact on growth, nutrient digestibility, health and antioxidant status in weaned piglets. PLoS One. 2018;13(3):e0192978. Epub 2018/03/09. doi: https://doi.org/10.1371/journal.pone.0192978. PMID: 29518093; PubMed Central PMCID: PMC5843174.

28. Jose NM, Bunt CR, Hussain MA. Comparison of Microbiological and Probiotic Characteristics of Lactobacilli Isolates from Dairy Food Products and Animal Rumen Contents. Microorganisms. 2015;3(2):198-212. Epub 2015/01/01. doi: https://doi.org/10.3390/microorganisms3020198. PMID: 27682086; PubMed Central PMCID: PMC5023236.

29. Reuben RC, Roy PC, Sarkar SL, Alam RU, Jahid IK. Isolation, characterization, and assessment of lactic acid bacteria toward their selection as poultry probiotics. BMC Microbiol.

2019;19(1):253. Epub 2019/11/14. doi: https://doi.org/10.1186/s12866-019-1626-0. PMID: 31718570; PubMed Central PMCID: PMC6852909.

30. de Man Jd, Rogosa d, Sharpe ME. A medium for the cultivation of lactobacilli. Journal of applied microbiology. 1960;23(1):130-5. doi: https://doi.org/10.1111/j.1365-2672.1960.tb00188.x.

31. Maragkoudakis PA, Zoumpopoulou G, Miaris C, Kalantzopoulos G, Pot B, Tsakalidou E. Probiotic potential of Lactobacillus strains isolated from dairy products. International Dairy Journal. 2006;16(3):189-99. doi: https://doi.org/10.1016/j.idairyj.2005.02.009.

32. Rocha-Ramírez LM, Hernández-Chiñas U, Moreno-Guerrero SS, Ramírez-Pacheco A, Eslava CA. Probiotic Properties and Immunomodulatory Activity of Lactobacillus Strains Isolated from Dairy Products. Microorganisms. 2021;9(4). Epub 2021/05/01. doi: https://doi.org/10.3390/microorganisms9040825. PMID: 33924561; PubMed Central PMCID: PMC8069045.

33. Amelia R, Philip K, Pratama YE, Purwati E. Characterization and probiotic potential of lactic acid bacteria isolated from dadiah sampled in West Sumatra. Food Science and Technology. 2020;41:746-52. doi: https://doi.org/10.1590/fst.30020.

34. Bazireh H, Shariati P, Azimzadeh Jamalkandi S, Ahmadi A, Boroumand MA. Isolation of Novel Probiotic Lactobacillus and Enterococcus Strains From Human Salivary and Fecal Sources. Front Microbiol. 2020;11:597946. Epub 2020/12/22. doi: https://doi.org/10.3389/fmicb.2020.597946. PMID: 33343539; PubMed Central PMCID: PMC7746552.

35. Nami Y, Vaseghi Bakhshayesh R, Mohammadzadeh Jalaly H, Lotfi H, Eslami S, Hejazi MA. Probiotic Properties of Enterococcus Isolated From Artisanal Dairy Products. Front Microbiol. 2019;10:300. Epub 2019/03/14. doi: https://doi.org/10.3389/fmicb.2019.00300. PMID: 30863379; PubMed Central PMCID: PMC6400110.

36. Abbasiliasi S, Tan JS, Bashokouh F, Ibrahim TAT, Mustafa S, Vakhshiteh F, et al. In vitro assessment of Pediococcus acidilactici Kp10 for its potential use in the food industry. BMC Microbiol.

2017;17(1):121. Epub 2017/05/26. doi: https://doi.org/10.1186/s12866-017-1000-z. PMID: 28535747; PubMed Central PMCID: PMC5442676.

37. Kumar S, Stecher G, Suleski M, Sanderford M, Sharma S, Tamura K. MEGA12: Molecular Evolutionary Genetic Analysis version 12 for adaptive and green computing. Mol Biol Evol. 2024;41(12). Epub 2024/12/22. doi: https://doi.org/10.1093/molbev/msae263. PMID: 39708372; PubMed Central PMCID: PMC11683415.

38. Sievers F, Higgins DG. Clustal Omega, accurate alignment of very large numbers of sequences. Methods Mol Biol. 2014;1079:105-16. Epub 2013/10/31. doi: https://doi.org/10.1007/978-1-62703-646-7_6. PMID: 24170397.

39. Rashid S, Alsayeqh AF, Akhtar T, Abbas RZ, Ashraf R. Probiotics: alternative to antibiotics in poultry production. 2023. doi: http://dx.doi.org/10.47278/journal.ijvs/2022.175.

40. Sornplang P, Piyadeatsoontorn S. Probiotic isolates from unconventional sources: a review. J Anim Sci Technol. 2016;58:26. Epub 2016/07/21. doi: https://doi.org/10.1186/s40781-016-0108-2. PMID: 27437119; PubMed Central PMCID: PMC4949924.

41. Ehrmann MA, Kurzak P, Bauer J, Vogel RF. Characterization of lactobacilli towards their use as probiotic adjuncts in poultry. J Appl Microbiol. 2002;92(5):966-75. Epub 2002/04/26. doi: https://doi.org/10.1046/j.1365-2672.2002.01608.x. PMID: 11972703.

42. Sharma Bajagai Y, Klieve A, Dart P, Bryden W. Probiotics in animal nutrition: production, impacts and regulation2016.

43. Divyakolu S, Chikkala R, Ratnakar KS, Sritharan V. Hemolysins of Staphylococcus aureus—an update on their biology, role in pathogenesis and as targets for anti-virulence therapy. Advances in Infectious Diseases. 2019;9(2):80-104. doi: https://doi.org/10.4236/aid.2019.92007.

44. Hou F, Tang J, Liu Y, Tan Y, Wang Y, Zheng L, et al. Safety Evaluation and Probiotic Potency Screening of Akkermansia muciniphila Strains Isolated from Human Feces and Breast Milk. Microbiol

Spectr. 2023;11(2):e0336122. Epub 2023/02/15. doi: https://doi.org/10.1128/spectrum.03361-22. PMID: 36786638; PubMed Central PMCID: PMC10103750.

45. Chen F, Zhu L, Qiu H. Isolation and probiotic potential of Lactobacillus salivarius and Pediococcus pentosaceus in specific pathogen free chickens. Brazilian Journal of Poultry Science. 2017;19:325-32. doi: https://doi.org/10.1590/1806-9061-2016-0413.

46. Shahbaz F, Muccee F, Shahab A, Safi SZ, Alomar SY, Qadeer A. Isolation and in vitro assessment of chicken gut microbes for probiotic potential. Front Microbiol. 2024;15:1278439. Epub 2024/02/13. doi: https://doi.org/10.3389/fmicb.2024.1278439. PMID: 38348194; PubMed Central PMCID: PMC10860760.

47. Prasad J, McJarrow P, Gopal P. Heat and osmotic stress responses of probiotic Lactobacillus rhamnosus HN001 (DR20) in relation to viability after drying. Appl Environ Microbiol. 2003;69(2):917-25. Epub 2003/02/07. doi: https://doi.org/10.1128/AEM.69.2.917-925.2003. PMID: 12571012; PubMed Central PMCID: PMC143580.

48. Hoque M, Akter F, Hossain K, Rahman M, Billah M, Islam K. Isolation, identification and analysis of probiotic properties of Lactobacillus spp. from selective regional yoghurts. 2010.

49. Shehata MG, El-Sohaimy SA, El-Sahn MA, Youssef MM. Screening of isolated potential probiotic lactic acid bacteria for cholesterol lowering property and bile salt hydrolase activity. Annals of Agricultural Science (Cairo). 2016;61(1):65–75. doi: https://doi.org/10.1016/j.aoas.2016.03.001.

50. Kandylis P, Pissaridi K, Bekatorou A, Kanellaki M, Koutinas AA. Dairy and non-dairy probiotic beverages. Current Opinion in Food Science. 2016;7:58-63. doi: https://doi.org/10.1016/j.cofs.2015.11.012.

51. Park S, Song J, Park MA, Jang HJ, Son S, Kim DH, et al. Assessing the Probiotic Effects of Pediococcus pentosaceus CACC616 in Weaned Piglets. Microorganisms. 2023;11(12). Epub 2023/12/23. doi: https://doi.org/10.3390/microorganisms11122890. PMID: 38138034; PubMed Central PMCID: PMC10746064.

52. Ruiz L, Margolles A, Sánchez B. Bile resistance mechanisms in Lactobacillus and Bifidobacterium. Front Microbiol. 2013;4:396. Epub 2014/01/09. doi: https://doi.org/10.3389/fmicb.2013.00396. PMID: 24399996; PubMed Central PMCID: PMC3872040.

53. Lin J, Sahin O, Michel LO, Zhang Q. Critical role of multidrug efflux pump CmeABC in bile resistance and in vivo colonization of Campylobacter jejuni. Infect Immun. 2003;71(8):4250-9. Epub 2003/07/23. doi: https://doi.org/10.1128/iai.71.8.4250-4259.2003. PMID: 12874300; PubMed Central PMCID: PMC165992.

54. Shokryazdan P, Kalavathy R, Sieo C, Alitheen N, Liang J, Jahromi M, et al. Isolation and characterization of Lactobacillus strains as potential probiotics for chickens. Pertanika J Tropical Agric Sci. 2014;37(1):141-57.

55. Shin MS, Han SK, Ji AR, Kim KS, Lee WK. Isolation and characterization of bacteriocin-producing bacteria from the gastrointestinal tract of broiler chickens for probiotic use. J Appl Microbiol. 2008;105(6):2203-12. Epub 2009/01/06. doi: https://doi.org/10.1111/j.1365-2672.2008.03935.x. PMID: 19120666.

56. Nallala VS, Sadishkumar V, Jeevaratnam K. Molecular characterization of antimicrobial Lactobacillus isolates and evaluation of their probiotic characteristics in vitro for use in poultry. Food Biotechnology. 2017;31:20-41. doi: https://doi.org/10.1080/08905436.2016.1269289.

57. Melara EG, Avellaneda MC, Rondón AJ, Rodríguez M, Valdivié M, Martínez Y. Characterization of Autochthonous Strains from the Cecal Content of Creole Roosters for a Potential Use as Probiotics. Animals (Basel). 2023;13(3). Epub 2023/02/12. doi: https://doi.org/10.3390/ani13030455. PMID: 36766343; PubMed Central PMCID: PMC9913217.

58. Vieco-Saiz N, Belguesmia Y, Raspoet R, Auclair E, Gancel F, Kempf I, et al. Benefits and Inputs From Lactic Acid Bacteria and Their Bacteriocins as Alternatives to Antibiotic Growth Promoters During Food-Animal Production. Front Microbiol. 2019;10:57. Epub 2019/02/26. doi:

https://doi.org/10.3389/fmicb.2019.00057. PMID: 30804896; PubMed Central PMCID: PMC6378274.

59. Oyewole OF, Maria CO, Tope PS, Funmi OO. In vitro Study of Potential Probiotic Lactic Acid Bacteria Isolated from The Gut of Chickens in Abeokuta, Nigeria. Alexandria Journal for Veterinary Sciences. 2018;58(1). doi: http://dx.doi.org/10.5455/ajvs.290499.

60. Kizerwetter-Swida M, Binek M. Selection of potentially probiotic Lactobacillus strains towards their inhibitory activity against poultry enteropathogenic bacteria. Pol J Microbiol. 2005;54(4):287-94. Epub 2006/04/08. PMID: 16599299.

61. Júnior W, Ferrari Í, Viana de Souza J, Silva C, Costa M, Dias F. Characterization and evaluation of lactic acid bacteria isolated from goat milk. Food Control. 2015;53. doi: https://doi.org/10.1016/j.foodcont.2015.01.013.

62. Spanggaard B, Huber I, Nielsen J, Sick EB, Pipper CB, Martinussen T, et al. The probiotic potential against vibriosis of the indigenous microflora of rainbow trout. Environ Microbiol. 2001;3(12):755-65. Epub 2002/02/16. doi: https://doi.org/10.1046/j.1462-2920.2001.00240.x. PMID: 11846769.

63. Nallala V, Sadishkumar V, Jeevaratnam K. Molecular characterization of antimicrobial Lactobacillus isolates and evaluation of their probiotic characteristics in vitro for use in poultry. Food Biotechnology. 2017;31(1):20-41. doi: https://doi.org/10.1080/08905436.2016.1269289.

64. Hummel AS, Hertel C, Holzapfel WH, Franz CM. Antibiotic resistances of starter and probiotic strains of lactic acid bacteria. Appl Environ Microbiol. 2007;73(3):730-9. Epub 2006/11/24. doi: https://doi.org/10.1128/aem.02105-06. PMID: 17122388; PubMed Central PMCID: PMC1800751.

65. Vidhyasagar V, Jeevaratnam K. Evaluation of Pediococcus pentosaceus strains isolated from Idly batter for probiotic properties in vitro. Journal of Functional foods. 2013;5(1):235-43. doi: https://doi.org/10.1016/j.jff.2012.10.012.

66. Sharma P, Tomar SK, Goswami P, Sangwan V, Singh R. Antibiotic resistance among commercially available probiotics. Food research international. 2014;57:176-95. doi: https://doi.org/10.1016/j.foodres.2014.01.025.

 Click here to access/download;Figure;Fig1.tif

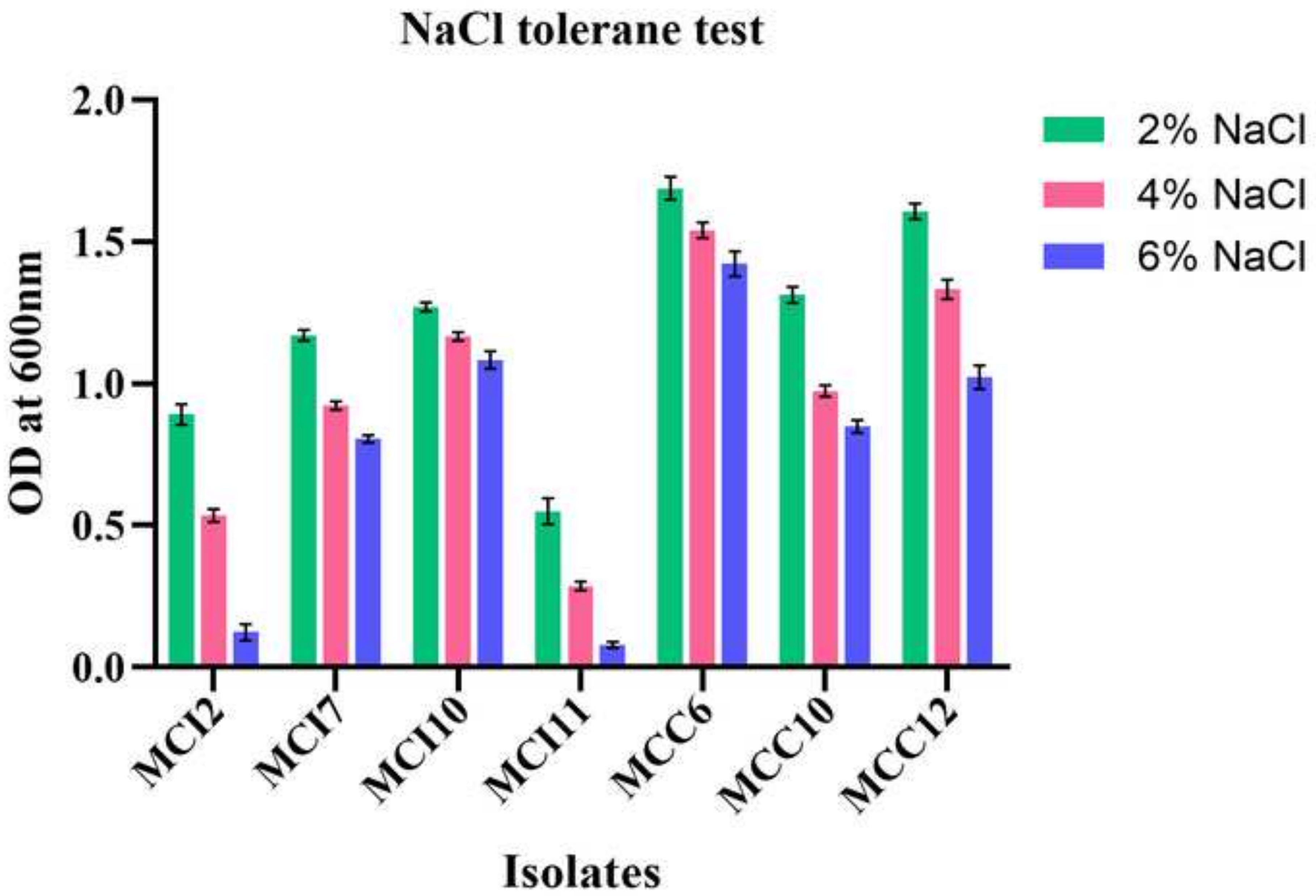

 Click here to access/download;Figure;Fig2.tif

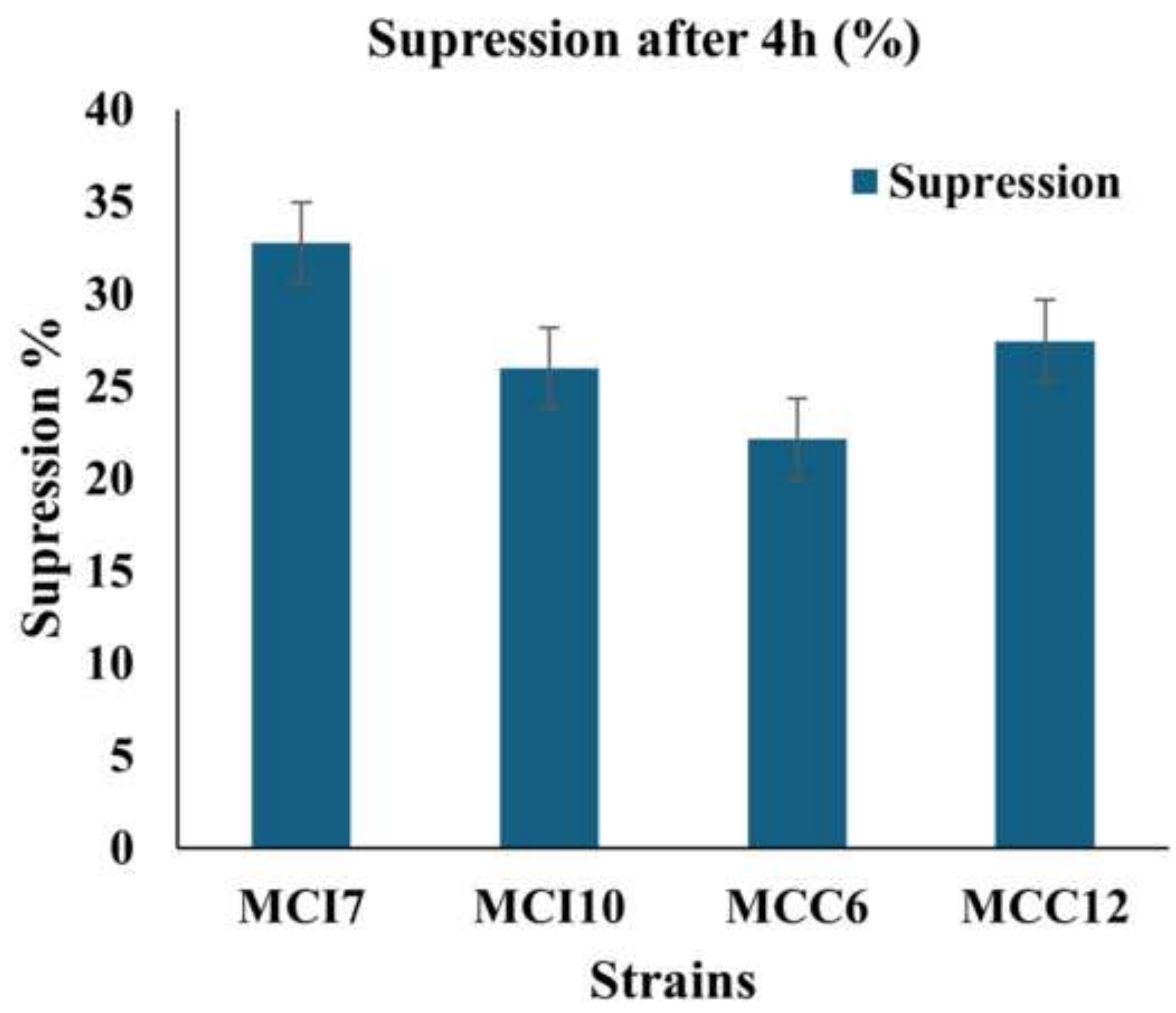

 Click here to access/download;Figure;Fig3.tif

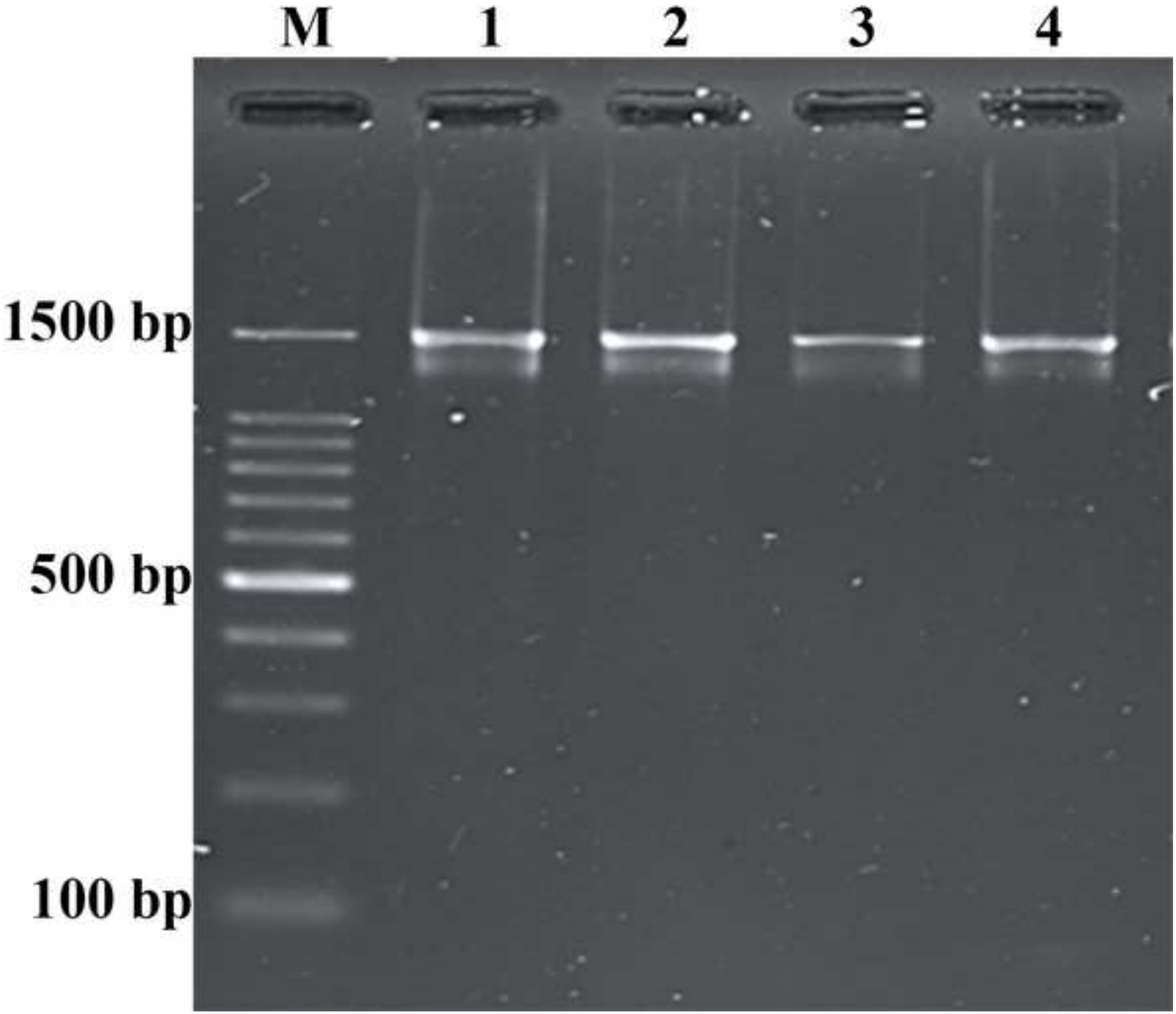

Figure Click here to access/download;Figure;Fig4.tif

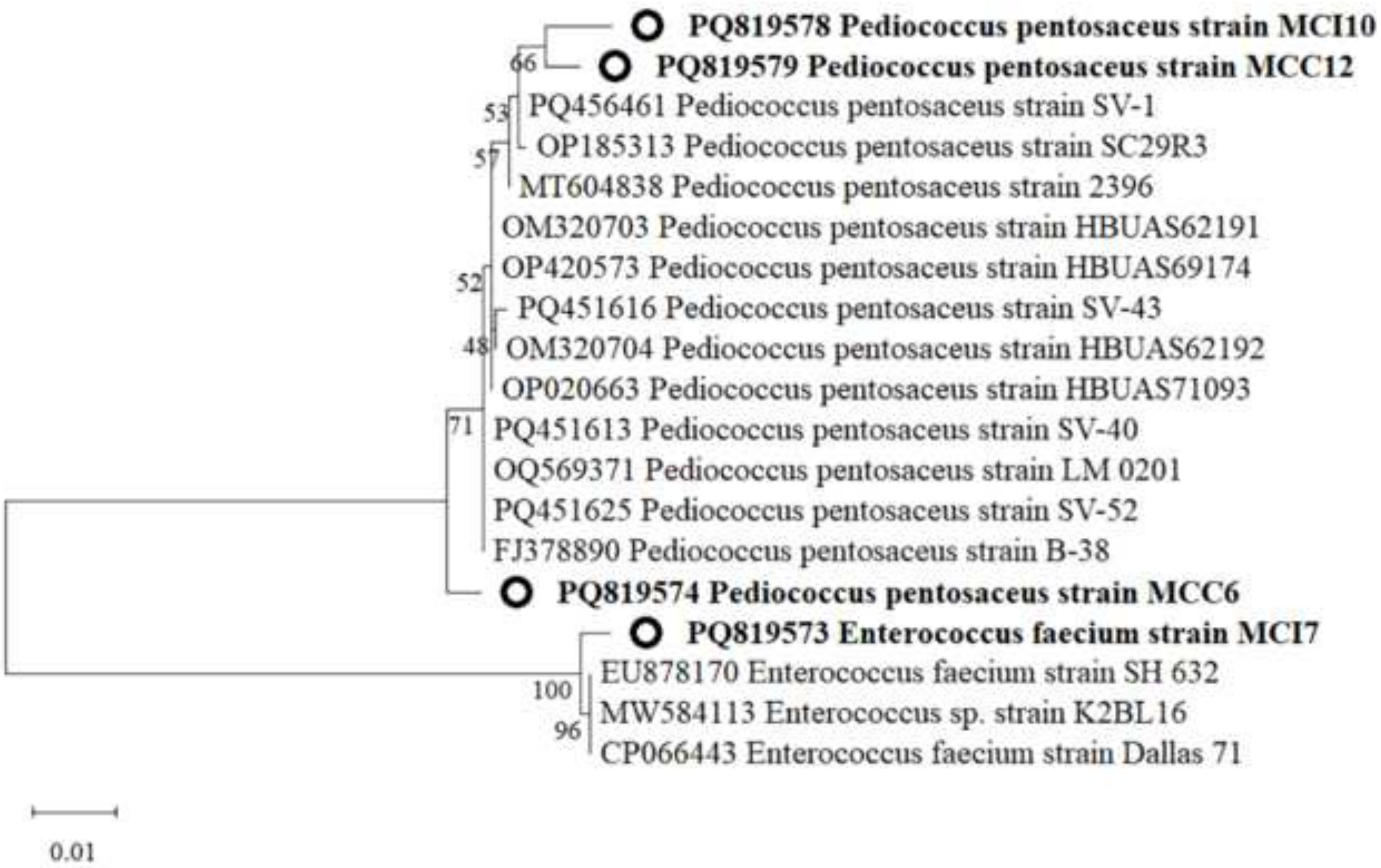

Click here to access/download
Supporting Information
S1_Fig.tiff

Click here to access/download
Supporting Information
S2_Fig.tif

Click here to access/download
Supporting Information
S3_Fig.tiff

Click here to access/download
Supporting Information
S4_Fig.tiff

Click here to access/download
Supporting Information
S1 Table.docx

Click here to access/download
Supporting Information
S2 Table.docx

Click here to access/download
Supporting Information
S3 Table.docx
