# Supplementary material for: In-vitro evaluation of probiotic potential of gut microbes isolated from retail chicken
Source: PLoS One. 2026 Jan 28;21(1):e0340981. doi: 10.1371/journal.pone.0340981 (PMC12851499; doi:10.1371/journal.pone.0340981)
Supplement: Attachment — Submitted filename: PLOS ONE comments.docx [file pone.0340981.s010.docx]

Current study is dealing with the prediction of probiotic potential of chicken gut associated bacteria via in-vitro analysis. Although we cannot recommend these bacteria as feed supplements without in-vivo experimentation, however, this study is forming basis for future in-vivo experimentation. So, it can be useful in future and has novelty. However, the number of assays performed for analysis of probiotic characteristics are not sufficient. Some modifications are needed to address before considering this article for publication.

**Minor comments:**

Methodology

1. L106: write GIT not the full term.
2. L121 and 122 are not methodology. Shift to Results section.
3. Which carbohydrates were selected for fermentation potential. Please write the names of tests.
4. Shift L120 after L114.
5. L146: Write abbreviation of colony forming units.
6. L152: Write the optical density with abbreviation and later use only abbreviation.
7. L159: Write Typhimurium as typhimurium.
8. L169-170, rephrase the sentence.
9. L201-202: rephrase and write correctly.
10. L205: remove with from with 20 each from intestine and cecum.
11. L309: Among these not of these.
12. L309, mention 4 as four.
13. L322: Mention lactic acid bacteria as LAB.

**Major comments:**

Methodology:

1. You did not perform serial dilution of enriched cultures. How did you get the countable number of colonies in this procedure? Kindly show the pictures of plates obtained after streaking of enriched cultures on MRS agar plates.
2. Shift molecular identification after the Morphological and biochemical characterization.
3. L164: Don’t use the term “probiotics” for these bacteria rather use fish gut born bacteria or gut associated bacteria.
4. L173-176: Describe the concentrations of antibiotics used.
5. Also perform the analysis of bacteria growth in simulated gastric medium and intestinal cell adhesion assay to confirm its probiotic characteristics. If it is easy then also perform cholesterol assimilation assay. But you should perform at least first two assays. Because number of assays documented here are not enough to confirm the probiotic potential of bacteria.

Results:

1. Figure 1 legend is not satisfactory. Describe it in detail and comprehensive way.
2. Rephrase the Table 1 caption.
3. Mention the p value in main paragraph not as foot note.
4. L243: Growth inhibition capability is not a correct term. Kindly replace with suppression.
5. L285-290: phylogenetic analysis description is very general and poor. Please discuss relation of each isolate with related genera and species with reference to bootstrap values mentioning the reliability of relation.
6. Figure 4 legend is poorly written. Kindly consult with published articles to improve your figure legends and table captions.

**Discussion**

1. Don’t mention human microbiota as it is irrelevant.
2. L319: bacteria did not show excellent tolerance to 6% NaCl.
3. L320: As you did not detect any stress tolerance associated protein so it is irrelevant to mention this sentence. Remove it.
4. L334: No reason to compare with pigs gut associated probiotics. There is a huge volume of literature on chicken gut probiotics. Kindly cite the relevant work.
5. Please cite more work from literature to compare the current study results.
6. In conclusion, describe one to two sentences which type of experimentation is needed in future perspectives of this study.
